# Molecular hallmarks of excitatory and inhibitory neuronal resilience to Alzheimer’s disease

**DOI:** 10.1186/s13024-025-00892-3

**Published:** 2025-10-01

**Authors:** Isabel Castanho, Pourya Naderi Yeganeh, Carles A. Boix, Sarah L. Morgan, Hansruedi Mathys, Dmitry Prokopenko, Bartholomew White, Larisa M. Soto, Giulia Pegoraro, Saloni Shah, Athanasios Ploumakis, Nikolas Kalavros, David A. Bennett, Christoph Lange, Doo Yeon Kim, Lars Bertram, Li-Huei Tsai, Manolis Kellis, Rudolph E. Tanzi, Winston Hide

**Affiliations:** 1 Harvard Medical School, Boston, MA USA; 2 Department of Pathology, Beth Israel Deaconess Medical Center, Boston, MA USA; 3 Computer Science and Artificial Intelligence Laboratory, MIT, Cambridge, MA USA; 4 Broad Institute of MIT and Harvard, Cambridge, MA USA; 5 Centre for Neuroscience, Surgery and Trauma, Blizard Institute, Queen Mary University of London, London, UK; 6 University of Pittsburgh Brain Institute, University of Pittsburgh School of Medicine, Pittsburgh, PA USA; 7 Picower Institute for Learning and Memory, MIT, Cambridge, MA USA; 8 Genetics and Aging Research Unit, MassGeneral Institute for Neurodegenerative Disease, Department of Neurology, Massachusetts General Hospital, Harvard Medical School, Charlestown, MA USA; 9 McCance Center for Brain Health, Massachusetts General Hospital, Boston, MA USA; 10 Genetics and Aging Research Unit, The Henry and Allison McCance Center for Brain Health, Department of Neurology, Massachusetts General Hospital, Boston, MA USA; 11 Medical School, University of Exeter, Exeter, UK; 12 Spatial Technologies Unit, Beth Israel Deaconess Medical Center, Boston, MA USA; 13 Rush Alzheimer’s Disease Center, Rush University Medical Center, Chicago, IL USA; 14 Department of Biostatistics, Harvard T.H. Chan School of Public Health, Boston, MA USA; 15 Lübeck Interdisciplinary Platform for Genome Analytics, Institutes of Neurogenetics and Cardiogenetics, University of Lübeck, Lübeck, Germany; 16 Department of Psychology, University of Oslo, Oslo, Norway; 17 Department of Brain and Cognitive Sciences, MIT, Cambridge, MA USA

**Keywords:** Cognitive resilience, Cognitive reserve, Alzheimer’s disease, Gene expression, Transcriptomics, Vulnerability, Genetics, Rare variants, Single-cell RNA sequencing, SST interneurons, E/I imbalance

## Abstract

**Background:**

A significant proportion of individuals maintain cognition despite extensive Alzheimer’s disease (AD) pathology, known as cognitive resilience. Understanding the molecular mechanisms that protect these individuals could reveal therapeutic targets for AD.

**Methods:**

This study defines molecular and cellular signatures of cognitive resilience by integrating bulk RNA and single-cell transcriptomic data with genetics across multiple brain regions. We analyzed data from the Religious Order Study and the Rush Memory and Aging Project (ROSMAP), including bulk RNA sequencing (*n* = 631 individuals) and multiregional single-nucleus RNA sequencing (*n* = 48 individuals). Subjects were categorized into AD, resilient, and control based on β-amyloid and tau pathology, and cognitive status. We identified and prioritized protected cell populations using whole-genome sequencing-derived genetic variants, transcriptomic profiling, and cellular composition.

**Results:**

Transcriptomics and polygenic risk analysis position resilience as an intermediate AD state. Only *GFAP* and *KLF4* expression distinguished resilience from controls at tissue level, whereas differential expression of genes involved in nucleic acid metabolism and signaling differentiated AD and resilient brains. At the cellular level, resilience was characterized by broad downregulation of *LINGO1* expression and reorganization of chaperone pathways, specifically downregulation of Hsp90 and upregulation of Hsp40, Hsp70, and Hsp110 families in excitatory neurons. MEF2C, ATP8B1, and RELN emerged as key markers of resilient neurons. Excitatory neuronal subtypes in the entorhinal cortex (ATP8B+ and MEF2C^high^) exhibited unique resilience signaling through activation of neurotrophin (BDNF-NTRK2, modulated by LINGO1) and angiopoietin (ANGPT2-TEK) pathways. MEF2C+ inhibitory neurons were over-represented in resilient brains, and the expression of genes associated with rare genetic variants revealed vulnerable somatostatin (SST) cortical interneurons that survive in AD resilience. The maintenance of excitatory-inhibitory balance emerges as a key characteristic of resilience.

**Conclusions:**

We have defined molecular and cellular hallmarks of cognitive resilience, an intermediate state in the AD continuum. Resilience mechanisms include preserved neuronal function, balanced network activity, and activation of neurotrophic survival signaling. Specific excitatory neuronal populations appear to play a central role in mediating cognitive resilience, while a subset of vulnerable interneurons likely provides compensation against AD-associated hyperexcitability. This study offers a framework to leverage natural protective mechanisms to mitigate neurodegeneration and preserve cognition in AD.

**Supplementary Information:**

The online version contains supplementary material available at 10.1186/s13024-025-00892-3.

## Graphical abstract

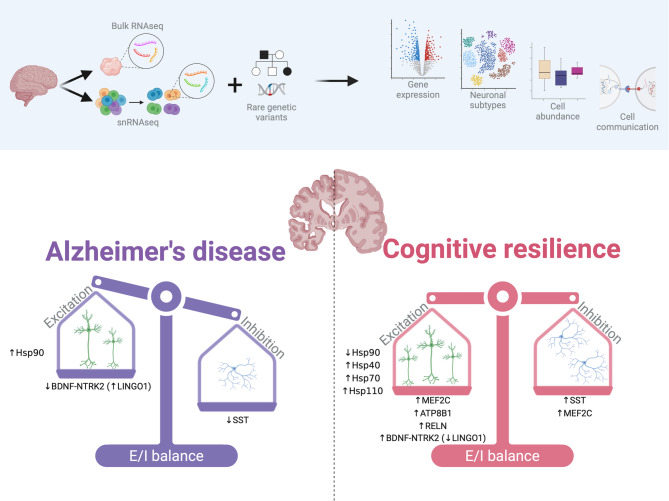

## Background

The search for effective disease-modifying treatments for Alzheimer’s disease (AD) has primarily focused on targeting β-amyloid (Aβ), with limited success. Historically, a definitive AD diagnosis has required postmortem observation of Aβ and tau neuropathology. However, some individuals maintain healthy cognitive function despite meeting neuropathological criteria for AD at autopsy, termed *cognitive resilience* (or cognitive reserve) [1–3]. In contrast, AD resistance describes individuals who do not develop AD neuropathology or cognitive decline, even in the presence of the strongest AD risk factor: advanced aging [1, 4]. Understanding natural protective mechanisms could be a potent approach to developing novel AD therapeutics beyond Aβ targeting. However, the precise molecular systems of protection against AD dementia via resilience or resistance mechanisms and their temporal relationship to AD pathogenesis remain poorly understood.

Genetic and high-throughput studies have identified molecular features associated with AD protection and risk [5–10]. These features are often attributed to resistance or vulnerability, but not resilience. Most healthy populations in these studies lack AD pathology and are not stratified by resilience phenotypes [11, 12]. It remains unclear whether protection occurs at the level of AD neuropathology (Aβ and tau) or AD dementia (neurodegeneration and cognitive decline).

Understanding how certain individuals prevent cognitive decline despite exposure to advanced pathology can lead to effective approaches tailored for presymptomatic stages of the disease. Recent genetic studies have shown that specific variants in *APOE* and *ATP8B1*, along with sex-linked loci, are associated with resilience [5–7, 13]. MEF2C promotes resilience in both humans and mouse models, likely by moderating excitatory transmission [14]. Single-cell genomics has revealed AD-associated vulnerability in specific cell types, such as somatostatin (SST+) GABAergic inhibitory neurons, and distinct gene expression programs that distinguish vulnerable individuals [8, 15–20]. Recent multiregion single-nuclei RNA sequencing (snRNAseq) analysis has revealed that *RELN*-expressing excitatory neuronal populations in the entorhinal cortex exhibit selective vulnerability to AD progression. The same study found that astrocytes express genes associated with resilience [19]. Maintaining cellular populations, such as SST+ inhibitory neurons or MEF2C+ and RELN+ excitatory neurons, appears to be integral to resilience. Contextualizing protective mechanisms such as resilience, which activate after the manifestation of pathological lesions, and resistance, which protect prior to pathology sets in, along the AD trajectory, is critical for translating these signals into actionable insights [3].

Despite their high potential for guiding therapeutic approaches for AD, the molecular and cellular underpinnings of resilience remain insufficiently described.

In this study, we systematically investigate molecular and cellular determinants of human AD resilience by integrating bulk RNA sequencing (RNAseq) and snRNAseq of different brain regions with rare genetic variants that confer AD risk and protection, to identify neuronal populations and cell-cell communication events that are important in resilience. Building on previous work that employed a continuous approach [19], we have now applied a discrete model to categorize resilience in the aging brain, paving the way for targeted interventions that could preserve cognitive function.

## Methods

This study aims to define molecular and cellular signatures of cognitive resilience, protection, and resistance against AD, by integrating genetics, bulk RNAseq, and single-nucleus RNAseq data across multiple brain regions.

### Human subjects

Clinical, pathologic and omic data are from participants in the Religious Orders Study or Rush Memory and Aging Project (ROSMAP). All ROSMAP participants enrolled without known dementia and agreed to detailed clinical evaluation and brain donation at death [21]. Both studies were approved by the Institutional Review Board of Rush University Medical Center. Each participant signed an informed consent, Anatomic Gift Act, and an RADC Repository consent. The evaluation includes 21 cognitive performance tests, 17 of which are summarized as global cognition and five cognitive domains [22]. The tests also inform on clinical diagnoses of AD dementia and mild cognitive impairment (MCI), and the reference no cognitive impairment (NCI) [23–25]. The neuropathologic assessment includes pathologic AD based on CERAD neocortical neuritic plaque estimates the severity and distribution of neurofibrillary tangles by Braak Stage; there are also quantitative measures of amyloid-β load and PHFtau tangle density derived by 8 brain regions [26–28]. Other neurodegenerative and vascular diseases are also documented [29, 30]. The brain omics data used in this study are described in detail below.

### Bulk RNAseq data analysis

#### Data retrieval and normalization

RNAseq profiles (*n* = 631) of dorsolateral prefrontal cortex (DLPFC) tissues of individuals from ROSMAP participants were accessed through the AMP-AD knowledge portal (syn2580853) [31, 32]. Read counts of samples with non-missing covariates were analyzed. The analysis was limited to genes with non-missing length and GC content, with at least one count per million read across all the samples. Samples were normalized for library size, GC content of genes, and genes’ lengths using the *Conditional Quantile Normalization* (CQN) method [33].

An iterative Principal Component Analysis (PCA)- based approach was used to determine significant non-biological, demographical, and technical confounding covariates (e.g., RNAseq quality metrics determined by the AMP-AD consortium using Picard Tools). The PCA-based analysis was performed using protocols previously described in the AMP-AD cross-cohort harmonization study [32]. Covariates that significantly correlated with the principal components of the gene expression data were selected for adjustment in downstream analysis.

The normalized RNAseq read counts were adjusted by determining the residual gene expression values from a linear model containing confounding covariates, implemented using the Limma-Voom method [34]. The final set of covariates for adjustment included: ​batch, sex, RNA Integrity number (RIN), %coding bases in each sample, %intergenic bases in each sample, post-mortem interval (PMI), age at death, and %pass-filter reads aligned.

#### Sample classification

Using levels of Aβ plaques and neurofibrillary tangles, and presence/absence of cognitive impairment, subjects with gene expression data available were classified into three major categories: AD (*n* = 187; diagnosis of AD dementia with no other cause of cognitive impairment; moderate/frequent plaques; Braak Stage III-VI), Resilient (RES, *n* = 68; “no cognitive impairment”; moderate/frequent plaques; Braak Stage III-VI; age > 80), Control (CTRL, *n* = 44; “no cognitive impairment”; sparse/none plaques; Braak Stage 0-II). An additional group of subjects was defined as Presymptomatic (PRE, *n* = 83, “mild cognitive impairment with no other cause of cognitive impairment”, moderate/frequent plaques; Braak Stage III-VI). All other subjects that did not fit the criteria described above were classified as “Other” (*n* = 249).

#### Differential gene expression analysis

Differentially expressed genes (DEGs) were determined by fitting a linear regression model adjusting for covariates using Limma-Voom. The following comparisons were performed, using moderated t-tests (implemented in Limma): ADvsRES, ADvsCTRL, RESvsCTRL, ADvsPRE, PREvsCTRL, and RESvsPRE. DEGs were determined by adjusting for confounding covariates (e.g., sex, age, and APOE status). *P*-values were adjusted for multiple comparisons using the False Discovery Rate (FDR) method [35]. Given that transcriptomic changes in DLPFC are more moderate compared to other brain regions [36], DEGs were determined using FDR < 0.1 and |log2FC| > log2(1.1) cut-offs (at least 10% difference in absolute expression).

#### Ordinal categorical analysis of loss of cognition

To determine genes associated with cognitive loss, we investigated an association between gene expression and loss of cognition using a Proportional Odds Model (POM). Gene expression levels were modeled as a function of cognitive status (ordinal categorical variable dependent on gene expression), adjusting for levels of Aβ pathology (plaques) and tau pathology (neurofibrillary tangles). The analysis was performed for each gene, implemented by a Proportional Odds Logistic Regression model using the VGAM library [37, 38]. All ROSMAP subjects with a cognitive status of “No cognitive impairment”, “Mild cognitive impairment with no other condition contributing to CI”, and “Alzheimer’s dementia with no other condition contributing to CI” (NINCDS/ADRDA “Probable AD”) were included in the analysis. Gene expression values were adjusted for confounding covariates (described above) prior to ordinal categorical analysis. Two-sided *p*-values associated with gene expression and other independent variables in the model were adjusted for multiple hypothesis comparison using the Bonferroni correction method.

#### Pathway activity analysis

To determine functional dysregulation events associated with AD and resilience, the PanomiR package was used to define differentially regulated pathways from the ROSMAP RNAseq data [39]. PanomiR uses a non-parametric rank-based summarization technique to generate pathway activity profiles from gene expression data [39–43]. In brief, the activity of a pathway in each sample represents the average squared ranks of the genes belonging to the pathway. PanomiR uses linear models via the Limma software package to compare pathway activity profiles between disease conditions and adjust for confounding covariates. In addition, PanomiR determines co-expressed modules of differentially regulated pathways by using network clustering algorithms and the Pathway Co-expression Network (PCxN) [42].

PanomiR was applied to the normalized ROSMAP gene expression data to map the activity of 1329 canonical pathways from the Broad Institute’s Molecular Signatures Database (MSigDB, C2, V6.2, July 2018). Dysregulated pathways were determined by comparing AD and resilient subjects using the *Limma* package [44], adjusted for confounding covariates determined in previous steps. Pathway-dysregulated *p*-values were adjusted for multiple-hypothesis testing using Storey’s q-value method [45]. Pathways with significant dysregulation (q-value < 0.1) between AD versus resilient subjects were mapped to PCxN. The mapping of dysregulated pathways to the PCxN network was performed using the default parameters provided in the PanomiR package, i.e., the network only contained edges corresponding to a correlation of 0.316 (sqrt(0.1)) with a significance threshold of FDR < 0.05, excluding nodes without any edges in the PCxN network. Clusters of dysregulated pathways were determined using the *Label Propagation* algorithm, implemented in the *igraph* package [46].

### Single-nuclei RNAseq data

#### Data processing

The generation of the single-nuclei RNAseq (snRNAseq) dataset covering multiple brain regions from 48 individuals from ROSMAP is described in the original publications [19, 47]. Clusters corresponding to major cell types were defined across brain regions as initially reported, subcell types were re-annotated for each brain region separately (described below). Counts for protein-coding genes were extracted from snRNAseq pre-processed data (filtered for mitochondrial and ribosomal RNA, and filtered for doublets, as described in [19] and reanalyzed independently for each brain region. Quality control (QC), additional filtering, normalization, and scaling were performed using the package *Seurat* [48](version 4.1.1) in R (version 4.1.2).

#### Clustering and annotations

Brain region-, cell type-specific subclusters (unsupervised) were generated using Harmony [49] (version 1.0, resolution = 0.5), with integration by subject. Cluster annotations were generated by a combination of reference mapping and manual curation. Cells from each brain region were mapped separately to two brain reference datasets, the 10x Whole Human Brain [50], and the Human MTG SEA-AD [20], using the web tool MapMyCells [51], and compared to the annotations previously described [19]. The mapping was performed using the Hierarchical algorithm. Final annotations of each cell subtype cluster were curated manually considering the results obtained from both references and marker genes, identified as described below.

#### Sample classification

Similarly to bulk RNAseq, using detailed phenotyping information from ROSMAP, subjects were classified as AD (Braak III-VI, CERAD “definite AD” or “probable AD”, and consensus cognitive diagnosis “Alzheimer’s dementia and no other cause of cognitive impairment”), RES (Braak III-VI, CERAD “definite AD” or “probable AD”, and consensus cognitive diagnosis “no cognitive impairment”), and CTRL (Braak 0-II, CERAD “no AD”, and consensus cognitive diagnosis “no cognitive impairment”). Subjects outside these criteria, classified as Other, were excluded from further analyses. We selected the dorsolateral prefrontal cortex (DLPFC) as the main focus for our initial investigations for consistency with bulk data, and we also investigated the entorhinal cortex (EC) and hippocampus (HC) as brain regions affected early in AD. Number of subjects and cells per group is shown in Table S11.

#### Differential gene expression analysis

Differential gene expression analyses were performed by implementing a statistical model by group using the MAST statistical framework [52](two-part generalized linear model), with a random effect for individual [53], integrated with the *Seurat* (version 4.1.1) workflow (*MAST* R package version 1.20.0). The following covariates were included in the differential expression analysis: sex, age at death, and number of unique molecular identifiers (UMIs). To find gene expression differences between the groups, the following comparisons were performed: ADvsRES, ADvsCTRL, and RESvsCTRL. All other parameters were set as default, except logfc.threshold (logfc.threshold = 0.05), with final sets of DEGs determined using |log2FC| >0.2. *P*-values were adjusted based on Bonferroni correction, as per Seurat’s recommendations (*satijalab.org/seurat/*), using all genes available in the dataset. Genes classified as mitochondrial genes were removed from the results tables and corresponding visualizations. In parallel, marker genes for each cell subtype, i.e., genes that define each subcluster, were identified via differential expression using the function *FindAllMarkers* from the package *Seurat* (version 4.1.1) in R (version 4.1.2) [48], with the following arguments: only.pos = TRUE, min.pct = 0.25, logfc.threshold = 0.25.

#### Gene ontology enrichment analysis

Gene ontology enrichment analysis and protein-protein interaction (PPI) enrichment analysis were performed using *Metascape* [54]. The expressed genes from each brain region-specific major cell type were used as the background list for each inquiry. The following pathway catalogs were queried: “GO Biological Processes”, “BioCarta Gene Sets”, “Canonical Pathways”, “Reactome Gene Sets”, “KEGG Pathway”, “WikiPathways”, “PANTHER Pathway”. Protein-protein interaction enrichment analysis was carried out using the following databases: STRING, BioGrid, OmniPath, and InWeb_IM. Up (log2FC > 0.2) and downregulated (log2FC < -0.2) genes (adj-*P* < 0.1) were investigated separately.

#### Differential composition analysis

To identify changes in cell composition (cell proportions) between AD, Resilient, and Control, we used a Dirichlet multinomial regression model, while accounting for the proportions of all of the other cell subsets within each major cell type. Changes in cell proportions and their associated *p*-values were determined using the *DirichReg* function in the *DirichletReg* (version 0.7-1) R package, and *P*-values were adjusted for multiple comparisons using the Benjamini-Hochberg (FDR) correction, as described by others [55]. Subclusters with a low number of cells (counts < 50 in either group) were removed from the analysis.

#### Intercellular communication

To investigate changes in intercellular communication between and within the various cell populations, we used the R package *CellChat* [56] (version 2.1.2) and followed its standard analysis pipeline with default settings.

### Whole genome sequencing data

To evaluate the cellular enrichment of genes from AD-associated rare variants, we have accessed summary statistics from a recent systematic analysis of rare variants associated with AD in two large whole-genome sequencing datasets [57]. The full analysis is described in the original publication. Briefly, single variant analysis and grouped rare variant analysis were performed in a family-based WGS dataset of 2247 subjects from 605 AD families and 1669 unrelated individuals. We have used two sets of results: (a) based on single rare variants from an extended supplementary Tables 12 and (b) based on grouped rare variants from supplementary Table 15 from the original publication [57]. Effect direction was identified from the Z-score for single rare variants and as the direction of the signal for the most significant single variant in the region-based analysis. Genetic variants found in asymptomatic family members were classified as ‘protective’, and genetic variants associated with AD were classified as ‘risk’ variants.

### Expression weighted cell type enrichment analysis

To investigate cellular enrichment of genes annotated from rare variants identified from WGS, we integrated snRNAseq data with rare variant-associated genes using expression-weighted cell type enrichment analysis using the R package *EWCE* (version 1.4.0) [58] with default settings, including Bonferroni correction of multiple comparisons. For computational feasibility, we partitioned the data using the R package *Caret* (representing each subject equally), and ran EWCE in 60% of the snRNAseq data, using 10,000 permutations. Subclusters with a low number of cells (counts < 50 in either of the groups) were removed from the analysis.

Genes from common variants were obtained from Bellenguez et al. 2022 (Tables 1 and 2 from the original publication) [11].

### Gene network prediction

To determine likely interactions and functional enrichment of marker genes expressed in SST+ vulnerable inhibitory neurons and genes associated with ‘protective’ and ‘risk’ variants overlapping genes that were expressed as markers in DFPLC:Inh1 were selected for network generation. A network of interacting node partners was generated using the STRING protein query feature of Cytoscape 3.10.2 set at default evidence.

### Immunostaining

Human brain tissue used for immunostaining experiments was collected at Beth Israel Deaconess Medical Center (BIDMC) upon autopsy (*n* = 16 subjects). Its use was approved by the BIDMC Institutional Review Board (IRB). Clinical and neuropathological phenotypes were used to assess each individual, including cognitive status proximate to death. Brains from age- and sex-matched de-identified individuals were classified into AD (Braak V-VI, CERAD “Moderate” or “Frequent”, and a clinical diagnosis of AD dementia; *n* = 6), Resilient (Braak V-VI, CERAD “Moderate”, and clinical records of absence of cognitive impairment; *n* = 4), and Control (Braak 0-I, CERAD “None” or “Sparse”, and clinical records of absence of cognitive impairment; *n* = 6). Subjects presenting comorbidities (frontotemporal lobar degeneration (FTLD), vascular dementia, Lewy body dementia, limbic-predominant age-related TDP-43 encephalopathy (LATE), and diabetes) were excluded. Blocks of formalin-fixed paraffin-embedded frontal lobe and mesial temporal lobe were sectioned (5 μm thickness) onto standard glass slides. Slides containing human brain tissue were processed for multiplex immunofluorescence and enzymatic immunohistochemistry.

### Multiplex immunofluorescence

Multiplex immunofluorescence (mIF) was achieved using the Opal 6-Plex Detection Kit (Akoya Biosciences, formerly Opal Polaris 7 Color IHC Automated Detection Kit). Staining was performed according to the manufacturer’s instructions. Frontal lobe sections, containing the DLPFC, were stained for β-tubulin (ab52623, Abcam, 1:200, 780 channel), GABA (PA5-32241, Thermo Fisher Scientific, 1:150, 690 channel), somatostatin (or SST, PA5-82678, Thermo Fisher Scientific, 1:200, channel 530), RBFOX1 (ab254413, Abcam, 1:200, channel 480), KIF26B (ab121952, Abcam, 1:100, channel 570), and Aβ (6E10, SIG-39300, BioLegend, 1:1000, channel 620). Mesial temporal lobe sections, containing the EC, were stained for β-tubulin (ab52623, Abcam, 1:200, 780 channel), NeuN (ab177487, Abcam, 1:150, channel 690), Reelin (or RELN, ab312310, Abcam, 1:150, channel 520), ATP8B1 (PA5-53839, Thermo Fisher Scientific, 1:150, channel 480), MEF2C (ab211493, Abcam, 1:150, channel 570), and Aβ (6E10, SIG-39300, BioLegend, 1:1000, channel 620). Tissue sections were imaged at 40x magnification on a PhenoImager HT (Akoya Biosciences) using whole slide scan settings. Brightness and contrast were adjusted using QuPath (version 0.4.3). Image processing was performed using *QuPath* [59], with cell detection based on DAPI being achieved using StarDist. Cell quantification was accomplished using a machine learning (ML) classifier within *QuPath*.

#### Analysis of single-cell protein quantifications from Qupath

Raw quantifications of marker intensities of 16 DLPFC samples (Control = 6, AD = 6, Resilient = 4) and 15 EC samples (Control = 6, AD = 6, Resilient = 5) were analyzed independently by region. Qupath quantifications were filtered first based on the upper and lower 1% percentiles of cell area, cell length and nucleus diameter; this approach is similar to that followed by Cheung et al. [60]. Subsequently, cells with autofluorescence values below the 1% percentile or higher than the 5% percentile were discarded from the analysis. In addition, cells from the EC were also filtered based on the mean intensity of NeuN, following an approach similar to that taken in single cell transcriptomic data to discard cells with high mitochondrial content. After filtering, each dataset was analyzed following the protocol for image-based spatial data analysis in Seurat 5.1.0 [61]. The subcellular organization of protein markers was preserved in downstream analysis by taking the mean intensity of each marker in each of three cellular compartments (nucleus, cytoplasm and membrane), and treating each compartment as an independent variable. For example, MEF2C quantifications resulted in three variables: MEF2C-Nucleus, MEF2C-Membrane and MEF2C-Cytoplasm. The spatially resolved data was imported into Seurat using the function *LoadAkoya.* The dataset was normalized and scaled using the centered log-ratio method, as recommended by the protocol. Principal component analysis was used to reduce the number of variables in each dataset by 50%. A UMAP embedding was obtained from the resulting principal components. The same number of principal components were used to find neighbors and perform clustering with a resolution of 0.4 in both datasets. Clusters were manually annotated by inspecting the expression of all the protein targets measured in each brain region. Cell types were named according to their RNA-derived counterparts whenever possible.

#### Comparison of cell proportions using protein-derived cell types

Once individual cell types were annotated, the comparison of cellular proportions was done following the same procedure described for the transcriptomic-based comparison, with a few modifications. The cellular proportions in the EC datasets are based on the pool of NeuN+ cells, which were removed from the beginning of the analysis with Seurat. In the DLPFC dataset, cells were not pre-filtered based on a neuronal marker; this was done after clustering. The population labeled as GABA- cells was discarded before the analysis of proportions. We opted for this approach due to the empirical distribution of GABA, which as opposed to the distribution of NeuN in the EC, had no obvious cutoff to split the neuronal population.

#### Analysis of marker co-expression

Co-expression of selected markers and their association with phenotypic data (e.g., disease group) were investigated using the processed mIF DLPFC and EC datasets separately. First, we calculated Pearson correlations between all pairwise combinations of markers in every cell population using the normalized intensities from Seurat. The resulting *P*-values were adjusted for multiple testing using the FDR method. For additional inspection of co-expression, an alternative series of linear models were used to account for additional covariates (general design formula: *response marker* ∼ *predictor marker* + *cell type* + *age* + *sex* + *disease group*) These formulations allow for reporting adjusted R-squared values, corresponding to the co-expression between the markers, as well as their associated *P*-values.

### Polygenic risk scores

To examine the genetic risk underlying each diagnostic group, polygenic risk scores (PRS) were calculated for each subject from ROSMAP with genetic data available [62] (*n* = 986) individually with PRSice-2 [63]using published effect sizes for 85 SNPs [11]. Each subject was classified as AD (*n* = 574), RES (*n* = 304), or CTRL (*n* = 108), as described for the bulk RNAseq analysis. Odds ratios were taken from published GWAS data [11] and applied to the samples used here to get individual PRS, and then compared across groups. All PRS were z-score normalized. A student’s T-test was used to compare sample groups, and *P*-values were adjusted for multiple testing using Bonferroni correction.

## Results

### Resilience as an intermediate state in the AD continuum

**Fig. 1:**
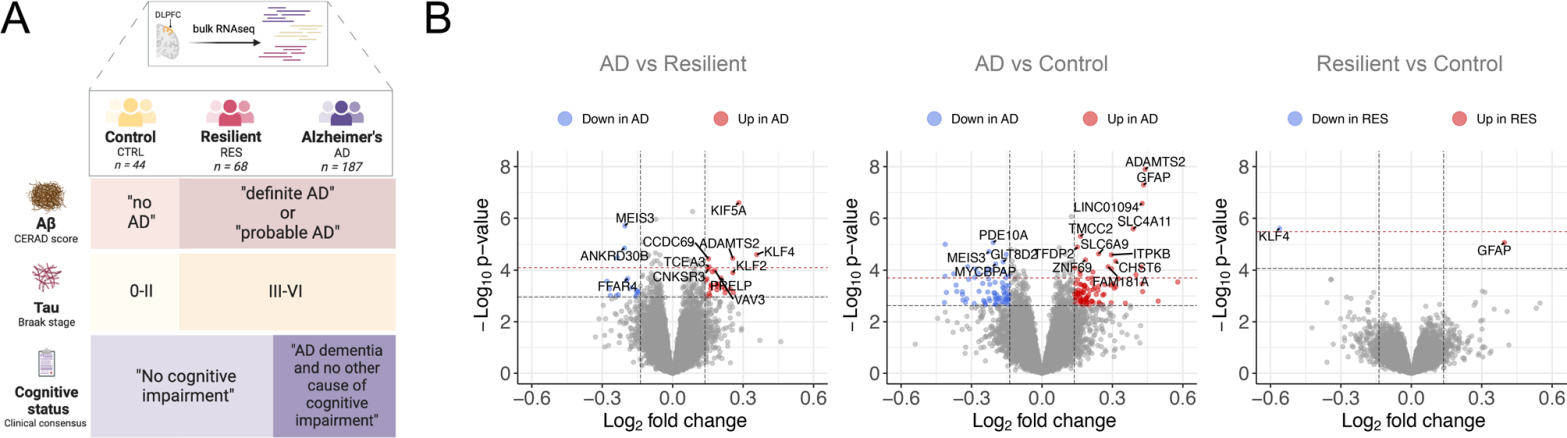
Transcriptomic signatures of cognitive resilience against AD pathology. (**A**) Overview of study design. Using levels of Aβ plaques and neurofibrillary tangles, and presence/absence of cognitive impairment, ROSMAP donors were classified into three major categories: Control (CTRL, CERAD “no AD”, Braak 0-II, and consensus cognitive diagnosis “no cognitive impairment”), Resilient (RES, CERAD “definite AD” or “probable AD”, Braak III-VI, and consensus cognitive diagnosis “no cognitive impairment”), and AD (CERAD “definite AD” or “probable AD”, Braak III-VI, and consensus cognitive diagnosis “Alzheimer’s dementia and no other cause of cognitive impairment”). (**B**) Volcano plots showing significantly (FDR-adj-*P* < 0.1) differentially expressed genes (DEGs) in AD compared to resilient individuals (ADvsRES), AD compared to controls (ADvsCTRL), and resilient compared to controls (RESvsCTRL). DEGs with log2FC < -log2(1.1) are highlighted in blue, and DEGs with log2FC > log2(1.1) are highlighted in red. The horizontal lines represent FDR-adjusted *P*-value = 0.1. Differential expression analysis performed implemented in the *Limma* package (moderated t-test). Statistical results are shown in Tables S1-S3. *n*_AD_ = 187, n_RES_ = 68, n_CTRL_ = 44. Figure created in part with BioRender.com

To define the molecular and functional signatures of resilience and to stratify them from those of AD and healthy individuals, we used published bulk RNAseq data from the dorsolateral prefrontal cortex (DLPFC) samples from the Religious Order Study and Memory and Aging Project (ROSMAP, *n* = 631) cohort [31]. Using levels of Aβ plaques and neurofibrillary tangles and the presence or absence of cognitive impairment [62], we classified ROSMAP participants into mutually exclusive groups: healthy controls, resilient, and AD (Fig. 1A and Figure S1). Samples that fell outside these strict group definitions were included in parallel investigations of genes linked to cognitive decline (**Methods**). To functionally characterize differentially regulated pathways in resilience and AD, we applied pathway-activity analysis (Extended results).

By comparing bulk RNAseq profiles between each group, we found a higher number of differentially expressed genes (DEGs) in AD compared to resilient individuals than in resilience compared to healthy controls (Fig. 1B, Tables S1-S3), reflecting more substantial transcriptomic changes in AD (see also Extended Results). Notably, the expression of only two genes, *KLF4* (KLF transcription factor 4; RESvsCTRL Log2FC = -0.56, adj-*P* = 0.04) and *GFAP* (encoding for glial fibrillary acidic protein; RESvsCTRL Log2FC = 0.40, adj-*P* = 0.07), was downregulated and upregulated, respectively, in resilient subjects compared to controls (Fig. 1B and Table S3). *GFAP* was also a top upregulated DEG in AD (Table S2, ADvsCTRL log2FC = 0.43, adj-*P* = 4.0 × 10^− 4^). Concordantly, single-cell expression of *GFAP* in the DLPFC was significantly upregulated in resilience (Extended Results). Taken together with the small number of DEGs, the increase in *GFAP* supports the view that resilient brains are molecularly positioned between healthy aging and AD. *KLF4*, a pro-inflammatory transcription factor in glial cells, exhibited an opposing expression pattern in AD and resilience (ADvsRES Log2FC = 0.36, adj-*P* = 0.035; RESvsCTRL Log2FC = -0.56, adj-*P* = 0.038), suggesting resilience-specific downregulation of *KLF4*, likely related to suppression of neuroinflammation [64].

Next, we assessed the extent to which transcriptomic shifts observed in resilience overlap with changes associated with cognitive impairment. We identified cognitive decline-associated gene expression changes using ordinal categorical regression (**Methods**) and compared these to resilience-associated DEGs to identify common signatures. Of the 54 genes significantly associated with cognitive impairment (54 genes with adj-*P* < 0.1; 46 genes with adj-*P* < 0.05, adjusted for pathology burden, Table S5), 20.4% (11 out of 43 DEGs with adj-*P* < 0.1; 4 out of 8 with adj-*P* < 0.05) were also differentially expressed in AD compared to resilience (Figure S2A and Table S5). Our results show that a considerable portion of resilience-associated DEGs are directly correlated with the events that lead to cognitive decline in AD.

To establish whether resilience is a distinct outcome of AD pathogenesis or an intermediate phenotype on the trajectory to AD, we compared AD polygenic risk scores (AD-PRS) in ROSMAP participants with whole-genome sequencing (WGS) [62] that we classified as healthy controls, resilient, and AD (**Methods**). In the resilient group, the AD-PRS, which evaluates risk prediction for AD considering AD-associated single-nucleotide polymorphisms (SNPs), was positioned between AD and controls (Figure S4). Because *APOE* and other age-of-onset-modifying variants were included in the analysis, this intermediate profile is most consistent with a delayed rather than absent transition toward symptomatic AD.

Thus, according to our results, resilience appears to reflect sustained preservation of cognition during a protracted pre-symptomatic phase of the AD continuum.

### Ubiquitous markers of cellular resilience

To characterize the cellular landscape of cognitive resilience, we assessed cell-type-specific signatures in published snRNAseq profiles from DLPFC, entorhinal cortex (EC), and hippocampus (HC) from the multiregion ROSMAP dataset (*n* = 48 subjects) [19]. As with the bulk RNAseq, we classified individuals into AD, resilient, and control groups, and carried out clustering and differential gene expression analyses (**Methods**, Figures S5-S7, Tables S11-S13). Major cell types were identified as previously described [19].

Individual cell types in the DLPFC, EC, and HC showed a consistently higher number of DEGs in AD compared to resilience than controls (Figure S8A, Table S12, ADvsRES compared to ADvsCTRL). Astrocytes, particularly in the DLPFC, exhibited more pronounced upregulation and downregulation of genes in AD (Figure S8B, Table S12). Excitatory neurons from all brain regions, as well as inhibitory neurons from the DLPFC and EC, also showed more pronounced gene expression changes in AD (Figure S8B, Table S12, ADvsRES compared to ADvsCTRL). Alongside the broad transcriptomic shifts detailed below, we observed changes in gene expression of established AD loci, including *GFAP*, *APOE*, and *PLCG2* (Extended Results).

**Fig. 2:**
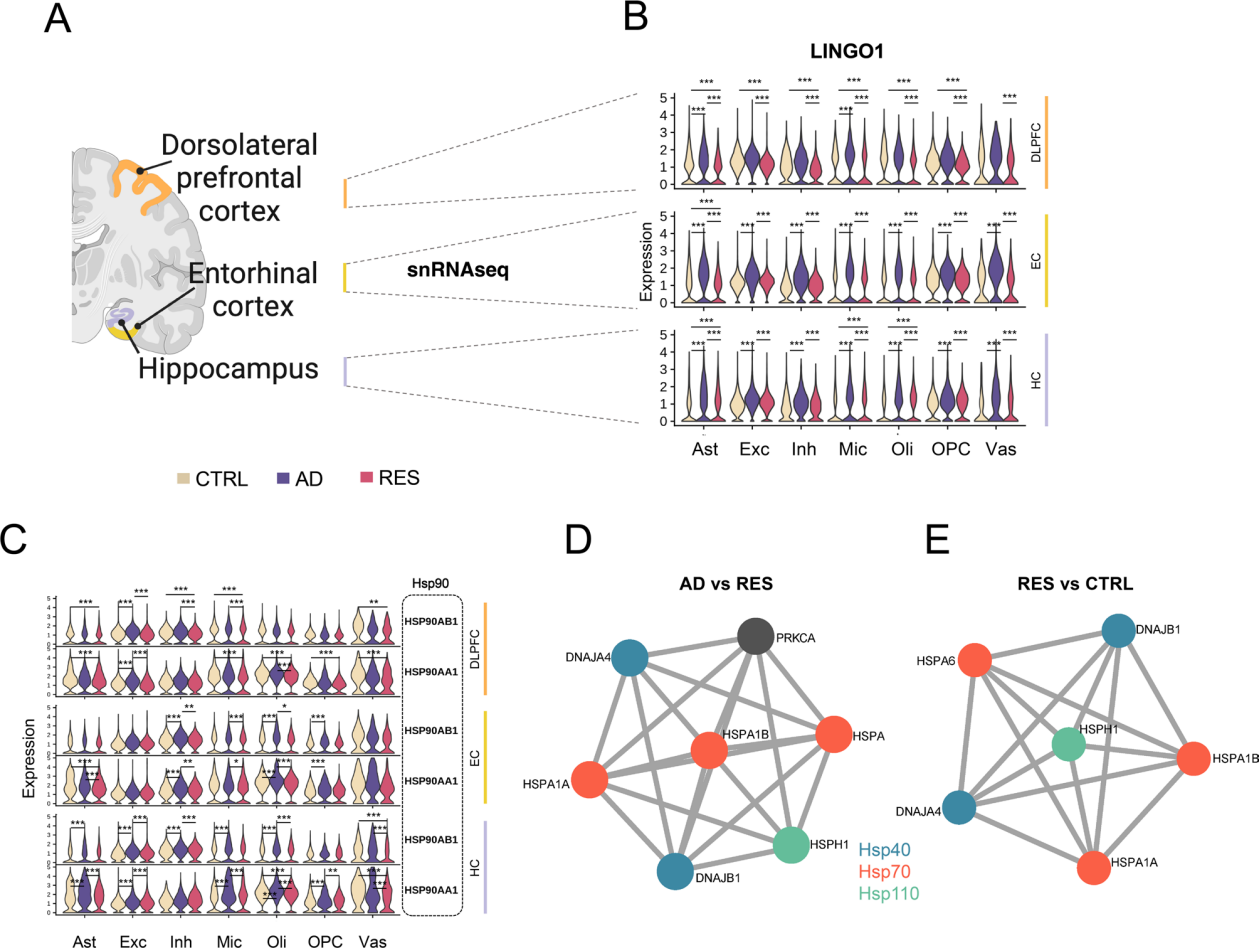
Cell-specific transcriptomic signatures of cognitive resilience against AD pathology. (**A**) Brain regions analyzed. Created with BioRender.com. (**B**) Down-regulation of *LINGO1* in cognitive resilience across different major cell types from each brain region (statistical results shown in Table S13). (**C**) Down-regulation of Hsp90 (heat shock protein 90) family members in cognitive resilience (statistical results shown in Table S13). (**D-E**) Protein-protein interaction (PPI) networks showing upregulation of members of the Hsp40, Hsp70, and Hsp110 families in resilient excitatory neurons. Molecular Complex Detection (MCODE) algorithm network clusters (modules) showing the subset of proteins that form physical interactions with at least one other member in the list, generated using *Metascape*. The protein networks were constructed based on physical interactions among all input gene lists. (**D**) PPI network analysis generated a single cluster from genes upregulated in excitatory neurons (DLPFC) in resilience compared to AD (ADvsRES). The three best-scoring terms by *p*-value from pathway and process enrichment analysis for this module were “chaperone cofactor-dependent protein refolding”, “‘de novo’ post-translational protein folding”, and “‘de novo’ protein folding” (Table S15). (**E**) Single PPI network cluster detected from genes upregulated in excitatory neurons (DLPFC) in resilience compared to controls (RESvsCTRL, Table S16). The three best-scoring terms by *p*-value from pathway and process enrichment analysis for this module were “chaperone cofactor-dependent protein refolding” (GO:0051085, Log10(P) = -13.2), “‘de novo’ post-translational protein folding” (GO:0051084, Log10(P) = -12.9), and “‘de novo’ protein folding” (GO:0006458, Log10(P) = -12.6). CTRL: Control, AD: Alzheimer’s disease, RES: Resilient. DLPFC: Dorsolateral prefrontal cortex, EC: Entorhinal cortex, HC: Hippocampus. *P*-values shown in **B**-**C** were derived from differential expression analysis performed using MAST in *Seurat* and adjusted using Bonferroni correction: * adj-*P* < 0.05, ** adj-*P* < 0.01, *** adj-*P* < 0.001. Log2FC, adj-P, and direction of change (first diagnostic group compared to the second group) shown in Table S13. Sample size distributions shown in Table S11

*LINGO1*, a negative regulator of BDNF-NTRK2-mediated neurotrophic signaling [65, 66] that has been implicated in axonal and synaptic loss [67], was consistently upregulated in AD but downregulated in resilience (Fig. 2B, Table S13). Relative to controls, *LINGO1* expression was ubiquitously decreased across cell types in DLPFC and EC tissue of resilient individuals (Table S13). Conversely, resilient HC astrocytes, microglia, and oligodendrocytes showed higher *LINGO1* levels (Table S13), indicating that the resilience-associated downregulation of *LINGO1* expression is largely cortex-specific. The concerted downregulation of *LINGO1* across cortical neurons and glia in resilience, in contrast to its upregulation in AD, points to a coordinated, circuit-level neurotrophin-dependent repair response and synaptic support.

Two members of the Hsp90 family of chaperones (HSP90AB1 and HSP90AA1), which are key regulators of tau folding and proteostasis [68, 69], showed consistent, downregulated expression in resilience across all three brain regions. Both transcripts were broadly higher in AD than resilience, with levels of *HSP90AB1* rising in most classes of neuronal and glial cells across the DLPFC, EC, and HC, and *HSP90AA1* increasing mainly in AD excitatory neurons and oligodendrocytes (Fig. 2C and Table S13). Compared to controls, resilient brains displayed an opposite pattern to AD: *HSP90AB1* levels were decreased in DLPFC astrocytes, inhibitory neurons, oligodendrocytes, and vascular cells, while *HSP90AA1* showed a decline in all non-neuronal cell types in DLPFC, EC astrocytes, and HC oligodendrocytes and vascular cells. Thus, downregulation of Hsp90 isoforms emerges as a cross-regional hallmark of cellular resilience.

Gene ontology (GO) enrichment analysis of upregulated and downregulated DEGs in major cell types from each comparison (ADvsRES, ADvsCTRL, and RESvsCTRL) identified multiple terms related to protein folding in resilience (Figure S10, “‘de novo’ protein folding” downregulated in AD compared to resilient, and “protein refolding” upregulated in resilient compared to controls). Protein-protein interaction (PPI) network analysis generated a single cluster from the list of upregulated genes in resilient DLPFC excitatory neurons compared to AD (ADvsRES, Fig. 2D, Table S15). This cluster was enriched in terms related to protein folding and included members of the families Hsp40 (*DNAJA4*, *DNAJB1*), Hsp70 (*HSPA1A*, *HSPA1B*, *HSPA6*), and Hsp110 (*HSPH1*). Concurrently, PPI analysis of upregulated genes in resilient excitatory neurons relative to controls (RESvsCTRL) produced one cluster enriched for “protein folding” ontology terms (Fig. 2E, Table S16), which included the same members and Hsp families. Our results are consistent with a recent study on an individual carrying a known pathogenic autosomal-dominant mutation in *PSEN2*, which showed pronounced elevation of Hsp70 family members and enrichment of protein-folding pathways in the cerebrospinal fluid [70]. In sum, in contrast with AD, although Hsp90 isoforms were downregulated in all regions and cell types in resilience, genes encoding other chaperones – Hsp40, Hsp70, and Hsp110 – were consistently upregulated in resilient neurons (Table S13), Taken together, these findings indicate complex transcriptomic dynamics in inhibitory and excitatory neurons that are distinct in resilience compared with AD and healthy controls.

### Inhibitory neurons exhibit resistance-associated phenotypes and are preserved in resilience

**Fig. 3:**
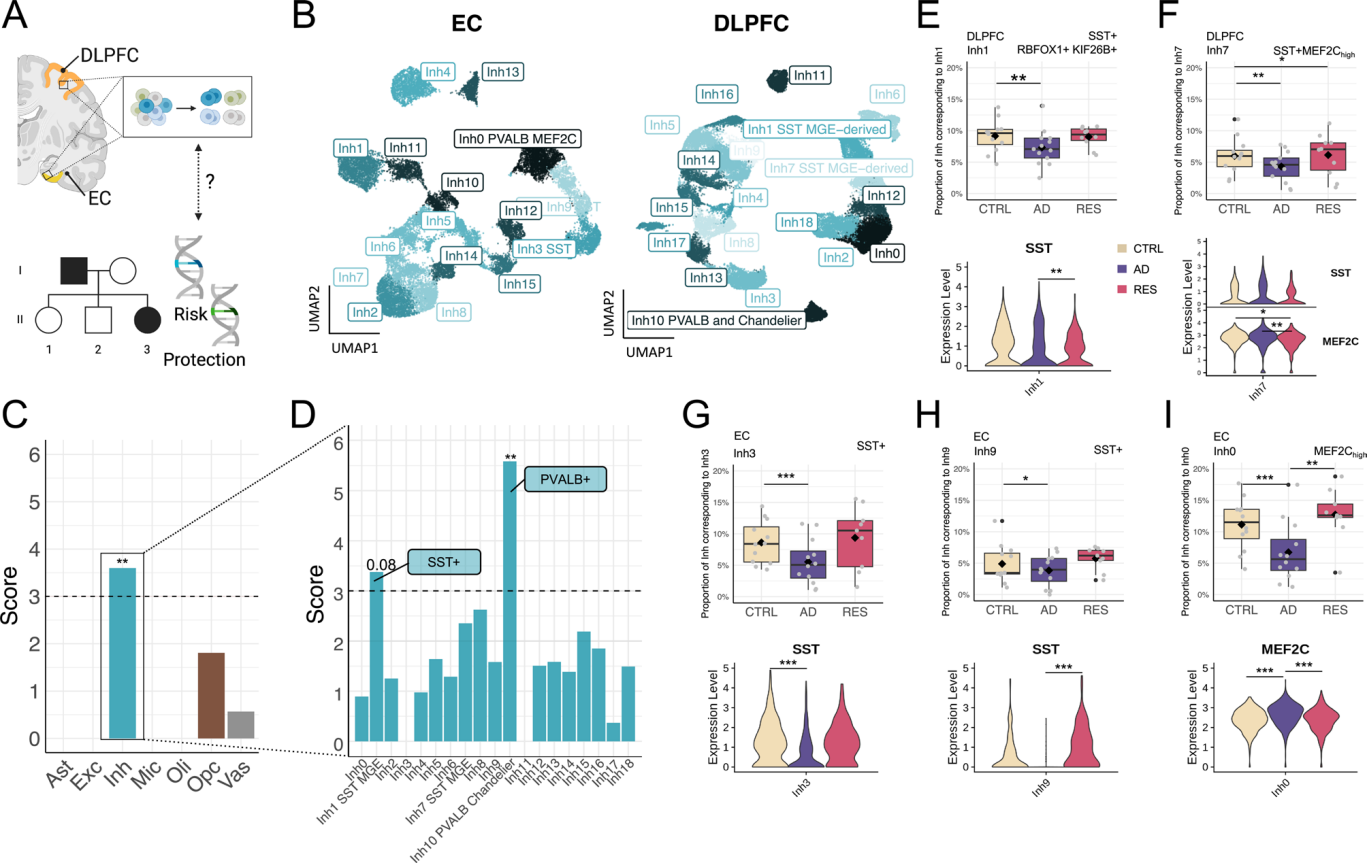
Inhibitory neurons as key players in protection against AD. (**A**) Brain regions shown in **B**-**I**. Created with BioRender.com. (**B**) UMAP embedding of inhibitory neurons from the EC (left) and DLPFC (right). Sample size distributions shown in Table S11. See Table S14 for detailed cluster annotations. (**C-D**) Cellular enrichment of genes annotated from protective rare variants in (**C**) major cell types (adj-*P* = 0.002) and (**D**) subtypes (adj-P_Inh1_ = 0.08, adj-P_Inh10_ = 0.007) of inhibitory neurons from the DLPFC, independently of diagnostic group. Expression-weighted cell type enrichment analysis performed using the R package *EWCE*, which calculates the probability of distribution of a gene list. Y-axis shows standard deviation from the bootstrapped mean. Stars denote Bonferroni-adjusted *P*-values (** adj-*P* < 0.01). (**E-I**) Distributions of cell proportion (top) and gene expression levels of marker genes (bottom) for the DLPFC *SST*+ (*RBOFX1*+ *KIF26B*+) Inh1 (**E**), *SST*+ *MEF2C*_high_ Inh7 (**F**), and the EC *SST*+ Inh3 (**G**), *SST*+ Inh9 (**I**), and *MEF2C*_high_ Inh0 (**J**) subpopulations. Stars shown in the box plots (cell proportions) reflect FDR-adjusted *P*-values from a Dirichlet multinomial regression model (Table S17), and in the violin plots (gene expression) refer to Bonferroni-adjusted *P*-values: * adj-*P* < 0.05, ** adj-*P* < 0.01, *** adj-*P* < 0.001

To define vulnerable and resilient cells, we examined the expression of genes associated with protection and risk across cell classes and contrasted the results with differential cell composition in our AD, resilient, and age-matched healthy classifications. Specifically, we applied Expression Weighted Cell Enrichment (EWCE) to the snRNAseq multiregional ROSMAP dataset (Fig. 3). Rare variants (single-nucleotide polymorphisms with minor allele frequency ≤ 1%) were selected from our recent whole-genome survey of families afflicted with AD [57, 71]. Rare genetic variants found in asymptomatic family members were classified as ‘protective’, and genetic variants associated with AD were classified as ‘risk’ variants (see **Methods** and **Limitations**). Common genetic variants were selected based on a recent study of approximately 800 K individuals [11].

Inhibitory neurons from the DLPFC and EC (Fig. 3) were significantly enriched for the expression of ‘protective’ genes associated with rare variants (Fig. 3C, Figure S11C), independently of group classification. This was also true for those in HC (Figure S11C). The expression of ‘risk’ genes associated with rare variants was also significantly enriched in inhibitory neurons as well as oligodendrocyte progenitor cells (OPCs) in all brain regions tested (Figure S11A-C), which we confirmed and validated in an independent DLPFC snRNAseq dataset [72] (Figure S11D). Risk genes associated with common variants [11] showed a strong expression bias in microglia and immune cells in all brain regions (Figure S11E), consistent with recent studies [73].

At the cell subtype level (Fig. 3B), two DLPFC inhibitory neuron subpopulations showed enrichment for protective gene expression (Fig. 3D), DLPFC:Inh1 (adj-*P* = 0.08) and DLPFC:Inh10 (adj-*P* = 0.007). Cell subtypes were identified from brain region-, cell type-specific subclusters, and annotated using two independent brain references (Fig. 3B and Table S14). Based on subtype-specific ‘marker genes’ (transcripts significantly enriched in a given subtype relative to all other cells, see **Methods**), we classified DLPFC:Inh1 as SST+ inhibitory neurons, and DLPFC:Inh10 as parvalbumin (PVALB)-expressing inhibitory neurons. Consistent results showing enrichment of protective genes in inhibitory neurons were replicated across several SST+ subtypes in an independent dataset [72] (Figure S11G). In the EC, SST+ EC:Inh3 interneurons also showed significant enrichment for protective variant-associated genes (Figure S11F). Therefore, cortical SST+ inhibitory neurons express genes associated with both protection and risk, suggesting these cell subtypes are important in the pathogenesis of AD.

To identify which inhibitory neuron classes are associated with resilience, we investigated changes in the composition of cell subtypes in each brain region according to group classification in the snRNAseq ROSMAP dataset (Dirichlet test, Table S17). Cell proportions for SST+ (RBFOX1+ KIF26B+) DLPFC:Inh1 and SST+ MEF2C_high_ DLPFC:Inh7, which were enriched for protective (Fig. 3D) and risk (Figure S11B) genes, respectively, showed a decrease in AD (Fig. 3E-F). *RBFOX1* and *KIF26B*, identified as protective genes harboring rare genetic variants, emerged as potential markers of protection-associated SST+ DLPFC:Inh1 interneurons. Indeed, we confirmed the co-expression of RBFOX1 and KIF26B in cortical neurons using multiplex immunofluorescence (mIF) applied to DLPFC sections from an independent cohort (Extended Results). In the EC, the AD-associated reduction in SST+ cells was replicated in the two SST+ EC subclasses, EC:Inh3 and EC:Inh9 (Fig. 3G-H), in parallel to a decrease in the proportion of MEF2C_high_ EC:Inh0 cells in AD (Fig. 3I). We observed lower levels of *SST* gene expression in EC inhibitory neurons in AD (Table S13, ADvsRES: 4th DEG ranked by *p*-value, log2FC = -0.52, adj-*P* = 3.54 × 10^− 29^, ADvsCTRL: log2FC = -0.41, adj-*P* = 1.79 × 10^− 22^). The proportion of PVALB+ DLPFC:Inh10 cells decreased in resilient individuals compared to controls (Figure S11H, adj-*P* = 0.02). Our findings suggest a role for MEF2C+ interneurons in resilience and confirm the vulnerability of SST+ neurons in AD. We hypothesize that PVALB+ interneurons may play a protective role in the pathogenesis of AD, but rather than promoting resilience, this role involves resistance to neuropathology.

To investigate the functional activity of resilience-associated pathways, we analyzed cell-cell communication events in the snRNAseq ROSMAP dataset, inferred from ligand-receptor (L-R) co-expression using *CellChat*. Resilient brains showed a greater number of significant L-R pairs across major cell classes and subpopulations in both DLPFC and EC compared to AD and control (Figure S15A–B), consistent with a compensatory or preserved communication response. The SST signaling pathway was downregulated in AD across all brain regions but upregulated in resilient individuals (Figure S16). In the EC, in contrast with resilience and controls, there was a complete loss of communication originating from the SST+ EC:Inh9 subtype in AD (Figure S17A), which could be explained at least in part by reduced expression of *SST* in this subpopulation (Figure S17B). The SST receptor SSTR2 was predicted to interact with SST in all diagnostic groups, whereas SSTR1 was involved in interactions between subclasses of inhibitory and excitatory neurons from the EC solely in resilient individuals (Figure S17B and Table S19). Both classes of SST+ inhibitory neurons were predicted to interact with multiple classes of excitatory neurons, including EC:Exc2, EC:Exc3, and EC:Exc5 (Figure S17A).

Our findings confirm prior evidence of preferential loss of SST+ interneurons in AD and, for the first time to our knowledge, show that SST signaling dynamics in the entorhinal cortex reflect the pattern of vulnerability seen in AD and preservation in resilience, positioning molecular inhibitory systems in resilience as an intermediate state along the AD trajectory.

### Excitatory neurons are key players in resilience

Analysis of differential gene expression from bulk DLPFC (Fig. 1B) in the snRNAseq ROSMAP dataset revealed that genes downregulated in AD compared to resilient individuals were disproportionately enriched in excitatory neurons (Figure S3A-B). Genes whose expression was associated with cognitive decline exhibited downregulation in excitatory neurons, particularly excitatory neurons from cortical layers L2-4 (DLPFC:Exc0 and DLPFC:Exc1) (Figure S3C-D). This suggests functional changes, including at the transcriptomic level, in excitatory neuronal subtypes may drive the cognitive impairment observed in AD.

**Fig. 4:**
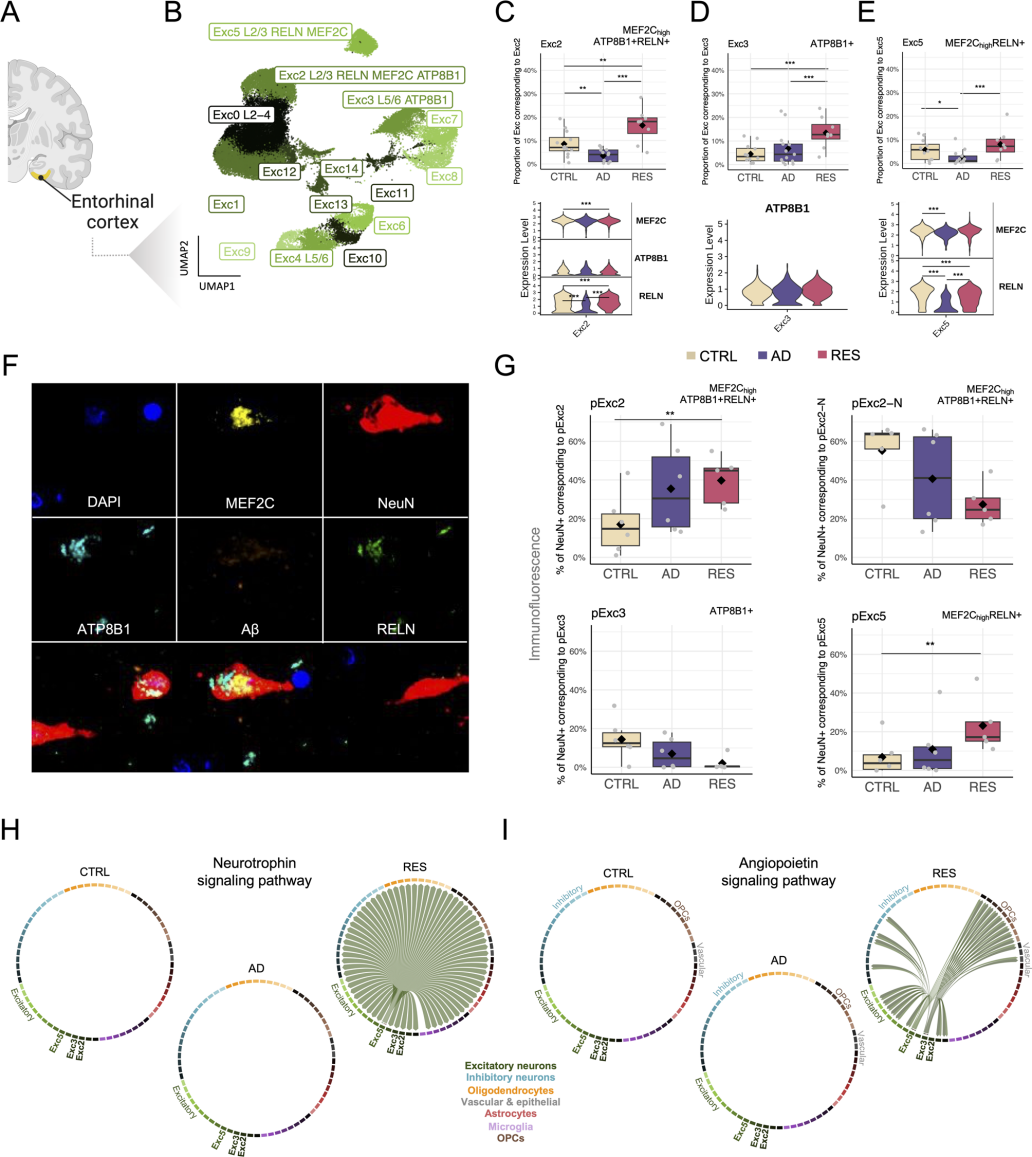
Excitatory neuronal subpopulations expressing MEF2C and ATP8B1 exhibiting resilient behavior. (**A**) Brain region shown in **B**-**I**. Created in part with BioRender.com. (**B**) UMAP plot showing the subclusters (‘subpopulations’) investigated in excitatory neurons from the EC, identified using the Harmony algorithm, in the ROSMAP cohort. Sample size distributions shown in Table S11. See Table S14 for detailed cluster annotations. (**C-E**) Cell proportion distributions (top) and gene expression levels of marker genes (bottom) for the *MEF2C*_high_ *ATP8B1*+ *RELN*+ EC:Exc2 subpopulation (**C**), *ATP8B1*+ EC:Exc3 subpopulation (**D**), and *MEF2C*_high_ *RELN*+ EC:Exc5 subpopulation (**E**). Stars shown in the box plots (cell proportions) reflect FDR-adjusted *P*-values from a Dirichlet multinomial regression model (Table S17), and in the violin plots (gene expression) refer to Bonferroni-adjusted *P*-values. (**F-G**) Protein staining in an independent cohort. Sample size distributions shown in Table S18. (**F**) Immunofluorescence representative pictures showing NeuN, RELN, MEF2C, ATP8B1, and Aβ in EC brain sections from a resilient subject. (**G**) Box plots showing cell proportion distributions for MEF2C^high^ATP8B1+ RELN+ (top; top left: positivity in non-nuclear compartments; top right: positivity in the nucleus), ATP8B1+ (bottom left), and MEF2C^high^ RELN+ (bottom right) neurons (NeuN+), identified by immunostaining. The subcellular organization of protein markers (nucleus, cytoplasm, and membrane) was considered in downstream analysis, treating each compartment as an independent variable. Data-driven single-cell clustering resulted in three variables: for MEF2C: Nucleus, Membrane, and Cytoplasm. For one of the clusters expressing MEF2C, ATP8B1, and RELN, the intensity quantifications were similar for MEF2C-Membrane and MEF2C-Cytoplasm, but distinct for MEF2C-Nucleus, thus reported here separately (pExc2: cytoplasm + membrane; pExc2-N: nucleus). Stars reflect FDR-adjusted *P*-values from a Dirichlet multinomial regression model. N_subjects_ = 17 (6 CTRL, 6 AD, 5 RES), N_cells_ = 81,549 (CTRL = 19,596, AD = 28,107, RES = 33,846) cells. (**H-I**) Chord diagrams displaying the neurotrophin (NT) signaling pathway (H) and angiopoietin (ANGPT) signaling pathway in EC cell subpopulations, predicted to change significantly (Figure S16) based on a cell-cell communication analysis of ligand-receptor interactions. * Adj-*P* < 0.05, ** Adj-*P* < 0.01, *** Adj-*P* < 0.001. CTRL: Control, AD: Alzheimer’s disease, RES: Resilient. DLPFC: Dorsolateral prefrontal cortex, EC: Entorhinal cortex, HC: Hippocampus

We explored the subpopulations of excitatory neurons in the ROSMAP snRNAseq dataset, which were identified from brain region- and cell type-specific subclusters and annotated using two independent brain references (Fig. 4B and Table S14). Our analysis revealed that multiple excitatory neuron subpopulations were overrepresented in the EC of resilient subjects (Fig. 4C-E), one of the first brain regions affected in AD. Two of these excitatory subtypes expressed *ATP8B1* as a marker gene (see **Methods**), and two expressed high levels of *MEF2C*. *MEF2C*_high_ subtypes also had a high expression of RELN (Reelin). Notably, *ATP8B1*, *MEF2C*, and *RELN* have been previously associated with cognitive resilience, either through genetic or gene expression studies [6, 18, 19, 74, 75]. EC:Exc2, annotated to cortical layers II-III, expressed both *ATP8B1* and *MEF2C* as marker genes. Consistently, two inhibitory neuron subclasses with *MEF2C* as a marker gene – the largest EC subcluster (EC:Inh0, Fig. 3I) and the DLPFC subtype DLPFCInh7 (Fig. 3F) – were over-represented in resilience, underscoring that *MEF2C*-expressing neurons are preferentially preserved during AD pathogenesis when cognition is maintained. *ATP8B1* expression was significantly upregulated in EC excitatory neurons of resilient subjects compared to controls (RESvsCTRL, Table S13). *MEF2C* expression showed no significant changes in the EC in major cell types but was significantly upregulated in AD and downregulated in resilience in multiple EC neuronal subtypes (Table S20). *MEF2C* was also significantly upregulated in AD and downregulated in resilience in hippocampal neurons in major cell types (Table S13) and subtypes (Table S20). In addition, we observed AD-associated depletion of two subtypes of excitatory neurons, one expressing *CDH9* (EC:Exc0) and the other *MEF2C* and *RBFOX1* (EC:Exc4), which did not exhibit significant changes in composition in resilience (Figure S14B). Both classes of excitatory neurons have recently been reported to be selectively vulnerable in AD [76].

To further explore excitatory neuronal subpopulations with a potential role in cognitive resilience, we mapped MEF2C, ATB8B1, and RELN proteins in EC sections from our independent cohort (*n* = 16 subjects; **Methods** and Extended Results) by staining for MEF2C, ATP8B1, RELN, NeuN, and Aβ (Fig. 4F). We followed the same procedure as in the DLPFC (Extended Results) to preprocess the resulting mIF data and identify and validate the presence of EC:Exc2, EC:Exc3, and EC:Ex5 (Figure S12C-F). As a quality control, we also reproduced the increase in Aβ+ cells in AD and resilient brains (Figure S14G). When examining the proportion of NeuN+ cells corresponding to the other two subpopulations of excitatory neurons, we reproduced the increased representation of MEF2C_high_ RELN+ (EC:pExc5) and MEF2C_high_ ATP8B1+ RELN+ (EC:pExc2) neurons (NeuN+) in resilient subjects that we had observed in the snRNAseq analysis (Fig. 4G). Changes in proportions of ATP8B1+ (EC:pExc3) cells could not be replicated with immunostaining (Fig. 4G).

To our surprise, we found two distinct cell clusters expressing MEF2C, ATP8B1, and RELN proteins, based on the mIF analysis, both equivalent to the EC:Exc2 from the snRNAseq ROSMAP dataset: (1) pExc2, expressing similar levels of MEF2C in all cellular compartments, and (2) pExc2-N, which expressed MEF2C in the nucleus (Figure S14H). Of the two, only pExc2 constituted a higher proportion of neurons in resilient subjects (Fig. 4G, pExc2); whereas the proportion of pExc-N cells was reduced in resilience (Fig. 4G, pExc2-N). Moreover, the levels of nuclear MEF2C were significantly higher in resilient than in control brains among pExc-N cells (Figure S14I). These results suggest a potential role for post-transcriptional or post-translational mechanisms in regulating the subcellular localization of MEF2C and its contribution to resilience.

Inspection of cell-cell communication in the resilience-associated excitatory subpopulations revealed two pathways that were unique to resilient EC excitatory neurons (i.e., were absent in control and AD): neurotrophin (Fig. 4H) and angiopoietin (Fig. 4I). For the neurotrophin signaling pathway, EC:Exc5 was labeled as the neuronal subtype of origin (or source), BDNF as the ligand, and NTRK2, SORT1 (sortilin 1), and NGFR (nerve growth factor receptor) as receptors. BDNF-NTRK2 signaling is critical for neuronal functions such as survival, morphogenesis, and plasticity [77]. Notably, the expression of *LINGO1*, the negative regulator of BDNF-NTRK2 is broadly downregulated in resilient subjects (Fig. 2B). This implies that the elimination of negative regulation by LINGO1 directly reshapes the integration of extracellular signals by specific excitatory neurons, promoting neuronal survival and glial support in resilient brains. The angiopoietin signaling pathway (Fig. 4I) had EC:Exc3 and EC:Exc5 as the source neuronal subclasses, ANGPT2 (angiopoietin-2) as the ligand, and TEK receptor tyrosine kinase and ITGA5:ITGB1 (integrin alpha-5/beta-1) as receptors. Because ANGPT2 regulates angiogenesis through TEK and integrin signaling [78], and promotes neuroprotection in a model of ischemic stroke [79], the ANGPT2–TEK/ITGA5:ITGB1 signaling axis from EC:Exc3/Exc5 to fibroblasts (EC:Fib) likely represents a resilience-linked angiogenic support mechanism.

Additional signaling pathways showing changes in resilience (Figure S16 and S18) with EC:Exc5 as source included ncWnt (non-canonical wingless-related integration site, targets: oligodendrocytes), periostin (target: EC:Inh1), BMP (bone morphogenetic protein, targets: subtypes from all major cell types), and TGF-β (transforming growth factor beta, target: ECFib). Of note, BMPs belong to the TGF-β superfamily, binding various TGF-β receptors, and are characterized by a mutually inhibitory crosstalk with canonical Wnt signaling [80]. Also, we observed a loss of TULP (tubby-like proteins) signaling between all three resilient excitatory neuronal subtypes (EC:Exc2, EC:Exc3, and EC:Exc5) and subtypes from multiple major cell types (Figure S18C). Signaling pathways observed to be lost in AD, with EC:Exc2, EC:Exc3, or EC:Exc5 as sources, are shown in Figure S19, and included, for example, KIT (or c-Kit) and EGF/EGFR signaling. Intriguingly, we noticed a shift of the EGF signaling pathway from EC:Exc2 as the source in controls to EC:Exc3 in resilience (Figure S19C, ligand: BTC; receptors: EGFR and ERBB4), which may represent a compensatory response in resilient brains.

Our results suggest that resilient brains selectively preserve and rewire MEF2C and ATP8B1-enriched excitatory neurons in the EC – by expanding their relative proportions, relocating MEF2C within the cell, and sustaining neurotrophin and angiopoietin signaling – and, so, position excitatory neurons as central players in resilience.

## Discussion

We have defined molecular determinants of resilience against AD dementia across brain tissues and within specific cellular populations. Resilient brains protect cognition through a combination of selective survival of inhibitory neurons in the DLPFC and increased representation of specific classes of excitatory neurons in the EC. These cells also upregulate the expression of genes associated with protein homeostasis and neuronal survival, downregulate the expression of genes related to neuroinflammation, and activate astrocytic responses to AD pathology.

Tissue-level transcriptomic differences in bulk RNAseq data are marked by upregulation of *GFAP* and downregulation of *KLF4* expression in resilience compared with healthy individuals (Fig. 1). GFAP, a key marker for reactive astrocytes [81], increases in expression with AD progression and may reflect early astrocytic activation in resilient brains. Resilience-dependent loss of *KLF4* expression, a nuclear transcription factor in microglia and endothelial cells, is reestablished in AD. Lipopolysaccharide (LPS) stimulation increases *KLF4* expression in microglia, while its knockdown reduces pro-inflammatory cytokines [64]. KLF4 levels also rise in microglia exposed to oligomeric Aβ42 [82], and its downregulation promotes axonal regeneration after optic nerve injury [83]. KLF4 may contribute to neuroinflammation in AD primarily by acting as a transcriptional regulator that amplifies pro-inflammatory signaling in microglia and astrocytes. Silencing of KLF4 in BV2 mouse microglial cells has been reported to attenuate oligomeric Aβ42-induced neuroinflammation by ameliorating the release of proinflammatory cytokines, such as tumor necrosis factor-a (TNF-α), interleukin (IL)-1β, IL-6, as well as inducible nitric oxide synthase (iNOS), and cyclooxygenase-2 (COX-2) [84]. Additionally, KLF4 targets are dysregulated in AD and linked to anti-inflammatory roles in brain endothelial cells [82]. Taken together, our data and prior reports position KLF4 as a pro-inflammatory hub whose resilience-associated suppression promotes neuroprotection, and re-emergence in AD contributes to neurodegeneration.

At the cellular level, we have identified pervasive reorganization of gene expression signatures associated with protein folding and degradation processes, which contribute to resilience across distinct cellular populations and brain regions (Fig. 2). This is characterized by widespread downregulation of Hsp90, contrasted by selective upregulation of Hsp40, Hsp70, and Hsp110 in excitatory neurons, exhibiting patterns opposite to those seen in AD. Differential regulation of these molecular chaperones, which all modulate tau [85–89], implies that cognitive resilience involves a distinct rearrangement of tau folding and clearance.

Further examination of cellular subtypes associated with resilience revealed cortical inhibitory neuronal populations that were enriched for the expression of AD protective and risk genes. Our results contribute to the growing body of evidence suggesting critical involvement of SST+ interneurons in the pathogenesis of AD, with *RBFOX1* and *KIF26B* (previously identified as candidate AD-protective genes from rare genetic variants [57, 71]) appearing as markers of protected SST+ interneurons (Figure S20 and Tables S21 and S22). Because SST interacts with Aβ to promote its degradation [reviewed in [90], SST is likely to exert a protective role by facilitating Aβ degradation and clearance in resilient brains. Moreover, we describe a population of resilience-associated inhibitory neurons expressing high levels of *MEF2C* (Fig. 3). MEF2C had been previously reported in excitatory neurons [14], but not in the context of inhibitory neurons. In AD dementia, cortical hyperexcitability is inversely correlated with overall cognition and executive function [91]. The degeneration of interneurons in AD likely disrupts the excitatory-inhibitory balance by impairing the inhibitory modulation of pyramidal neurons, destabilizing neuronal networks, and ultimately leading to hyperexcitability, which contributes to the cognitive deficits observed in AD. In contrast, our observations in inhibitory neurons from resilient brains suggest that excitatory-inhibitory balance is maintained in resilience.

Balanced network resilience appears to rely on coordinated adaptation in both inhibitory and excitatory neurons, as shown by our finding of an excitatory neuronal basis for resilience (Fig. 4). *ATP8B1*, *MEF2C*, and *RELN* emerged as key marker genes within these resilient subpopulations (Exc2, Exc3, and Exc5 in EC), with *ATP8B1* expression showing significant upregulation in resilient subjects compared to controls. This upregulation is consistent with previous findings associating *ATP8B1* and *MEF2C* with cognitive resilience in AD [6, 74]. *ATP8B1* expression was primarily upregulated in excitatory neurons from the EC, whereas *MEF2C* expression displayed significant upregulation in AD and downregulation in resilience across multiple neuronal subtypes from both the EC and HC. Beyond excitatory neurons, *MEF2C* was also a marker of resilience-associated subpopulations of inhibitory neurons in the EC and DLPFC. Our findings suggest that the role of MEF2C in cognitive resilience may extend beyond excitatory neurons, possibly contributing to the maintenance of the excitatory-inhibitory balance in key brain regions such as the EC.

Cell-cell communication analysis highlighted neuronal cell interactions in resilience, particularly through neurotrophin and angiopoietin signaling pathways. BDNF and its receptor NTRK2 (neurotrophin pathway) are essential for neuronal survival, synaptic plasticity, and neurogenesis [77]. BDNF and NTRK2 co-expression was uniquely active only in resilient subjects, suggesting a protective role in AD. Protective rare variants are associated with *NTRK2* and its interaction partner, *NGFR*. *LINGO1*, a negative regulator of BDNF-NTRK2 (Figure S21), was upregulated in AD across cell types but not in resilient brains. LINGO1 has been shown to enhance β-secretase cleavage of amyloid precursor protein (APP), increasing the generation of Aβ [65, 66, 92]. Thus, downregulation of LINGO1 may contribute to cognitive resilience by limiting the deposition of Aβ. Anti-LINGO1 antibodies improve cognition, promote neurogenesis and synaptic protection, and preserve GABAergic interneurons in AD mice [67, 93]. Our study is the first to report resilience-associated downregulation of *LINGO1*, which is largely cortex-specific. Therefore, therapeutic inhibition of LINGO1 could foster neuroprotection and cognitive resilience.

The angiopoietin signaling pathway, ANGPT2, TEK, and integrins ITGA5:ITGB1 were uniquely active in resilient subjects. ANGPT2 supports neuroprotection in ischemic stroke models [79]. Angiopoietin signaling from excitatory neurons to matrix-secreting fibroblasts suggests a neuron-driven, pro-healing environment that may contribute to preserving cognitive function in the face of AD pathogenesis. Therefore, ANGPT2-TEK signaling originating from excitatory neurons could be a neuroprotective target in AD.

**Fig. 5:**
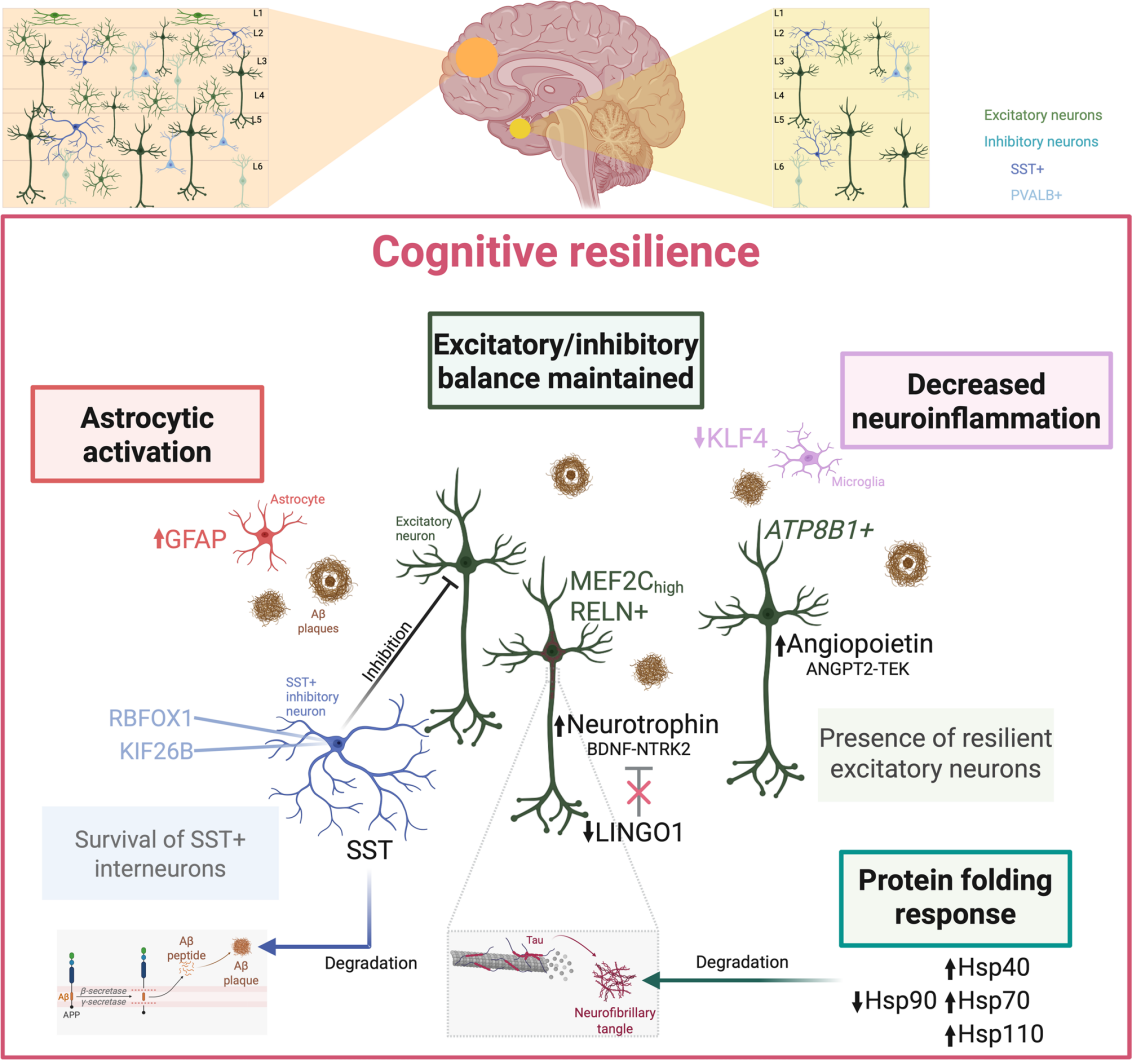
A functional model of resilience. Our model proposes that cognitive resilience is driven by the maintenance of the excitatory/inhibitory neuronal balance (dark green), sustained by resilient excitatory neurons expressing *MEF2C* and *ATP8B1*. These neurons engage in resilience-relevant signaling pathways, including neurotrophin (BDNF-NTRK2), modulated by the downregulation of *LINGO1*, and angiopoietin (ANGPT2-TEK). Protein folding and degradation processes are reorganized in resilience, with increased expression of Hsp40, Hsp70, and Hsp110 in excitatory neurons and downregulation of Hsp90, enhancing the degradation of pathological tau (mint green). *SST*+ inhibitory neurons, typically vulnerable in AD, are preserved in resilience, including subpopulations expressing *RBFOX1* and *KIF26B* (blue), contributing to the balance of neuronal excitation. Additionally, SST release from these neurons promotes the degradation and clearance of pathological Aβ. In terms of glial response, resilience shows astrogliosis marked by increased *GFAP* in astrocytes (red), a feature shared with AD. However, it contrasts with AD by exhibiting a reduction or absence of microglial activation, characterized by decreased *KLF4* expression, leading to reduced neuroinflammation (pink). Figure created with BioRender.com

Together, our data support a unified model of cognitive resilience that centers on coordinated neuronal (excitatory-inhibitory balance) and glial responses (astrocytic activation and decreased neuroinflammation by microglia), as well as adaptive protein-folding and clearance mechanisms (Fig. 5).

At the circuit level, SST+ interneurons (RBFOX1+, KIF26B+) are selectively preserved and promote Aβ degradation via SST release, and, together with resilient MEF2C+/RELN+ and ATP8B1+ excitatory neurons, maintain excitatory-inhibitory balance. In the resilient response to neuronal insults driven by AD pathogenesis, LINGO1 is downregulated, which in turn releases BDNF inhibition [94, 95], leading to autophosphorylation of NTRK2 [96]. This activates neurotrophin signaling cascades that promote neuronal survival, growth, and differentiation, likely including ERK5, which, in the nucleus, phosphorylates MEF2C and MEF2A, thereby activating their transcriptional activity. The expression of MEF2C, which is located in the nucleus of resilient neurons in our study, is associated with cognitive ability. MEF2C may play a significant role in cognitive resilience against AD by regulating genes whose expression is critical for neuronal survival, synaptic plasticity, and reduction of hyperexcitability [14], thereby sustaining pro-survival cascades in both excitatory and inhibitory neurons. MEF2C has been shown to modulate KLF4 [97], whose gene expression we identified as downregulated in resilient brains, likely representing decreased neuroinflammation. Finally, our results indicate that resilient excitatory neurons activate angiopoietin signaling targeted at vascular and epithelial cells, possibly promoting healing responses in vulnerable tissue. Collectively, these interconnected events define a regulated state in cortical regions that delays the transition from asymptomatic pathology to AD dementia.

### Limitations

Definitions of the (molecular) resilience phenotype in AD vary substantially across studies [18, 19, 98–101]. In this study, we adopted a discrete yet stringent definition of resilience, selecting individuals with advanced amyloid and tau pathology at death but preserved cognitive function. To stratify individuals who may eventually develop dementia if they live long enough, continuous longitudinal changes in cognition would be more appropriate in future studies, when possible. Without this approach, we were unable to capture resilience as deviations from expected cognitive trajectories, including moderate levels of resilience. Despite adjusting every analysis for age, sex, and APOE ε4 status, the unequal distribution of these factors across ROSMAP groups may still introduce residual bias, underscoring the need for validation in larger, demographically balanced cohorts. Additionally, we have not considered how other pathologies and their readouts, such as neuroinflammatory markers, contribute to dementia and resilience.

Other limitations of our study include the inherent challenges of working with rare genetic variants, which were identified through a familial genetic analysis and whole genome sequencing. Due to their rarity, these variants have not achieved genome-wide significance, which limits their statistical power. This limitation was partially addressed in our previous study by validation in an independent case-control WGS dataset [57]. Nonetheless, we have demonstrated that using a rank-based approach to select the most significant variants, followed by systems analysis, yields informative biological functions and cell-type-biased expression patterns.

### Future directions

To fully define and understand resilience and resistance to AD, future studies should map the activity of specific cell types to their precise spatial locations and integrate molecular determinants of resilience and cell-cell communication networks at the sites of activity. By understanding these relationships, we can pinpoint which cells and mechanisms should be targeted for protection to halt AD progression. Equally important is exploring these processes at the subcellular level, particularly within synapses, where early degenerative changes often occur. The role of synaptic health in resilience is critical, as synaptic dysfunction is one of the earliest hallmarks of AD [102]. Future work should additionally investigate how sex differences impact resilience to AD, particularly given the disproportionate burden of AD in women. Sex-specific pathways may play a role in neuroinflammatory regulation, cognitive decline, and overall disease progression. Addressing these dimensions will provide a more comprehensive understanding of resilience mechanisms and offer new insights for therapeutic interventions.

## Conclusions

Our study advances the understanding of cognitive resilience by revealing specific molecular and cellular processes that may protect against cognitive decline in the trajectory of AD pathogenesis. We report molecular and cellular hallmarks of cognitive resilience in AD, emphasizing the preservation of excitatory/inhibitory balance as critical to cognitive resilience.

Through integrative transcriptomic and single-cell analyses, we have uncovered global reorganization of protein folding and degradation pathways, highlighted by selective upregulation of Hsp40, Hsp70, and Hsp110 families and downregulation of Hsp90 in resilient excitatory neurons. Key excitatory neuronal subpopulations overrepresented in resilient brains, including neurons expressing ATP8B1, MEF2C, and RELN in the entorhinal cortex, demonstrated resilience-associated neurotrophin (in MEF2C_high_ RELN+ neurons) and angiopoietin signaling (in ATP8B1+ and MEF2C^high^ RELN+ neurons) mediated by the BDNF-NTRK2 and ANGPT2-TEK pathways, respectively. LINGO1 downregulation emerged as a novel mechanism promoting cognitive preservation.

The identification of resilient subpopulations of excitatory and inhibitory neurons, as well as key glial cell responses, exposes potential novel strategies that could emulate natural resilience processes to protect against AD dementia and offers new avenues for the prevention of neurodegeneration and cognitive decline.

## Supplementary Information

Below is the link to the electronic supplementary material.

Supplementary Figure 1 to 21

Supplementary Table: **Table S1**: Differentially expressed genes in AD compared to cognitive resilience in bulk DLPFC tissue. Differential gene expression analysis was performed between AD and resilient subjects (ADvsRES) using ROSMAP bulk RNAseq data. Significant differential expression was determined using adjusted *p*-value (FDR) < 0.1 (highlighted in red) and |FC| >1.1 criteria. *P*-values were determined using a linear regression model accounting for confounding covariates such as sequencing batch, RNA integrity number, and postmortem interval. The column “Direction” shows significant upregulation (“UP”) or downregulation (“DOWN”) in AD versus resilience as defined above. The column “Unique ADvsRES” reports genes that were differentially expressed in ADvsRES and not in ADvsCTRL or RESvsCTRL. The column “Cognitive loss” reports genes also associated with loss of cognition (related with Table S5). **Table S2**: Differentially expressed genes in AD compared to cognitively healthy controls in bulk DLPFC tissue. Differential gene expression analysis was performed between AD and control subjects (ADvsCTRL) using ROSMAP bulk RNAseq data. Significant differential expression was determined using adjusted *p*-value (FDR) < 0.1 (highlighted in red) and |FC| >1.1 criteria. *P*-values were determined using a linear regression model accounting for confounding covariates such as sequencing batch, RNA integrity number, and postmortem interval. The column “Direction” shows significant upregulation (“UP”) or downregulation (“DOWN”) in AD versus controls as defined above. **Table S3**: Differentially expressed genes in resilient subjects compared to cognitively healthy controls in bulk DLPFC tissue. Differential gene expression analysis was performed between resilient and control subjects (RESvsCTRL) using ROSMAP bulk RNAseq data. Significant differential expression was determined using adjusted *p*-value (FDR) < 0.1 (highlighted in red) and |FC| >1.1 criteria. *P*-values were determined using a linear regression model accounting for confounding covariates such as sequencing batch, RNA integrity number, and postmortem interval. The column “Direction” shows significant upregulation (“UP”) or downregulation (“DOWN”) in resilience versus controls as defined above. **Table S4**: Differentially expressed genes in AD compared to subjects classified as presymptomatic in bulk DLPFC tissue. Differential gene expression analysis was performed between AD and presymptomatic subjects (ADvsPRE) using ROSMAP bulk RNAseq data. Significant differential expression was determined using adjusted *p*-value (FDR) < 0.1 (highlighted in red) and |FC| >1.1 criteria. *P*-values were determined using a linear regression model accounting for confounding covariates such as sequencing batch, RNA integrity number, and postmortem interval. The column “Direction” shows significant upregulation (“UP”) or downregulation (“DOWN”) in AD versus presymptomatic as defined above. **Table S5**: Genes associated with loss of cognition from bulk RNAseq data. The association of gene expression profiles with loss of cognition was analyzed using a proportional odds model (POM) for ordinal categorical data analysis applied to ROSMAP bulk RNAseq data. Gene expression levels were adjusted for confounding covariates prior to performing POM. The POM analysis was applied to the subjects with either no cognitive impairment, mild cognitive impairment, or AD dementia. The POM analyzed cognitive impairment as a function of gene expression and pathology status (plaque and tangle stages). *P*-values, adjusted for multiple hypothesis comparison using the Bonferroni correction method (column “adj.P.Val”), represent the significance of the association of each gene with cognitive status. The column “Direction” denotes whether the expression of a gene positively or negatively correlates with loss of cognition. The column “ADvsRES” denotes genes that were also differentially expressed between AD and resilient subjects, “ADvsRES dir” reports the direction in AD compared to the resilient group (related with Table S1), and “Change consistency” reports the consistency between the two analyses. **Table S6**: Dysregulated pathways in AD compared to cognitive resilience in bulk DLPFC. Differential pathway activity analysis was performed between AD and resilient subjects based on ROSMAP bulk RNAseq data. Significant pathway dysregulation was determined using Storey-adjusted *p*-value (q-value) < 0.1. *P*-values were determined using a linear regression model that accounts for confounding covariates such as sequencing batch, RNA integrity number, and postmortem interval. **Table S7**: Unsupervised clusters of dysregulated pathways in AD compared to cognitive resilience from bulk DLPFC. Cluster membership for each of the 99 dysregulated pathways in ADvsRES (related to Table S6) following unsupervised clustering. Modules of dysregulated pathways in ADvsRES were determined by mapping to the pathway co-expression network followed by the Label Propagation clustering algorithm. The column “cluster” represents the unsupervised clusters assigned by the described analysis. The analysis was performed using the PanomiR package with default parameters. **Table S8**: Dysregulated pathways in AD compared to cognitively healthy controls in bulk DLPFC. Differential pathway activity analysis was performed between AD and control subjects based on ROSMAP bulk RNAseq data. Significant pathway dysregulation was determined using Storey-adjusted *p*-value (q-value) < 0.1. *p*-values were determined using a linear regression model accounting for confounding covariates such as sequencing batch, RNA integrity number, and postmortem interval. **Table S9**: Results for the pathway activity analysis in resilient individuals compared to subjects classified as presymptomatic in bulk DLPFC. Differential pathway activity analysis was performed between RES and presymptomatic subjects (RESvs PRE) based on ROSMAP bulk RNAseq data. Significant pathway dysregulation was determined using Storey-adjusted *p*-value (q-value) < 0.1. *P*-values were determined using a linear regression model that accounts for confounding covariates such as sequencing batch, RNA integrity number, and postmortem interval. **Table S10**: Results for the pathway activity analysis in AD compared to presymptomatic subjects in bulk DLPFC tissue. Differential pathway activity analysis was performed between AD and presymptomatic subjects (ADvs PRE) based on ROSMAP bulk RNAseq data. Significant pathway dysregulation was determined using Storey-adjusted *p*-value (q-value) < 0.1. *P*-values were determined using a linear regression model that accounts for confounding covariates such as sequencing batch, RNA integrity number, and postmortem interval. **Table S11**: Number of subjects and number of cells per group defined in this study for snRNAseq data. **Table S12**: Distributions for differentially expressed genes per comparison, identified from SnRNAseq data for each major cell type in each brain region investigated. Differential gene expression analyses were performed per major cell type for each brain region by implementing a statistical model by group using the MAST statistical framework in *Seurat*, after removing non-variable genes. *P*-values were adjusted using the Bonferroni correction, as recommended for the R package *Seurat*. Significant differential expression was determined using adjusted *p*-value (adj-P) < 0.1 and log2FC > 0.2 or log2FC < -0.2 (|FC| >1.1) criteria. The number of downregulated differentially expressed genes (DEGs) in the first group compared to the second group are highlighted in red, and the number of upregulated DEGs in the first group compared to the second group are highlighted in blue. **Table S13**: Selected differentially expressed genes for major cell types. Differential expression summary results for each brain region across the three comparisons (ADvsRES, ADvsCTRL, and RESvsCTRL) for genes discussed in the text. Differential gene expression analyses were performed per major cell type for each brain region by implementing a statistical model by group using the MAST statistical framework in *Seurat*, after removing non-variable genes. *P*-values were adjusted using the Bonferroni correction (adj-P), as recommended for the R package *Seurat*. Significant differential expression was determined using adjusted adj-*p* < 0.1 and log2FC > 0.2 or log2FC < -0.2 (|FC| >1.1) criteria. NS: not significant. Direction “NONE” refers to log2FC below our threshold. Extended results tables are available on Synapse (Synapse ID *syn63686123*). **Table S14**. Cell annotations for cell subtypes for each brain region. Cluster annotations were generated using the web tool MapMyCells using the Hierarchical algorithm. **Table S15**: GO enrichment results for the single PPI module of upregulated genes in resilient DLPFC excitatory neurons compared to AD. Performed with Metascape, considering the following databases: STRING, BioGrid, OmniPath, and InWeb_IM. **Table S16**. GO enrichment results for the single PPI module of upregulated genes in resilient DLPFC excitatory neurons compared to controls. Performed with Metascape, considering the following databases: STRING, BioGrid, OmniPath, and InWeb_IM. **Table S17**: Results for cell proportion analysis per major cell types for each brain region investigated. Changes in cell composition between groups for each cell subpopulation (subclusters) were detected using a Dirichlet multinomial regression model, while accounting for the proportions of all of the other cell subsets within each major cell type. *P*-values were adjusted for multiple comparisons using FDR correction (adj-P). Green: adj-*P* < 0.05, yellow: adj-*P* < 0.01, red: adj-*p* < 0.001. **Table S18**: Phenotypic and clinical characteristics of the BIDMC cohort. Human brain tissue used for immunostaining experiments was collected at Beth Israel Deaconess Medical Center (BIDMC) upon autopsy. Samples were tested for multiple pathologies, including TDP-43. Donors presenting comorbidities, including diabetes, were excluded. **Table S19**: Ligand-receptor results from cellchat for the SST signaling pathway in the EC. Source cell subtypes included EC:Inh3 and EC:Inh9 in control and resilient subjects, but in AD only EC:Inh3 and EC:Inh11 were identified as sources for the SST pathway in AD. The receptor SSTR1 was only observed in resilience. **Table S20**: Selected differentially expressed genes for cell subtypes. Differential expression summary results for each brain region across the three comparisons (ADvsRES, ADvsCTRL, and RESvsCTRL) for genes discussed in the text. Differential gene expression analyses were performed per cell subtype for each brain region by implementing a statistical model by group using the MAST statistical framework in *Seurat*, after removing non-variable genes. *P*-values were adjusted using the Bonferroni correction (adj-P), as recommended for the R package *Seurat*. Significant differential expression was determined using adjusted adj-*p* < 0.1 and log2FC > 0.2 or log2FC < -0.2 (|FC| >1.1) criteria. NS: not significant. Direction “NONE” refers to log2FC below our threshold. **Table S21**: Interaction partners of RBFOX1 identified as rare variant-associated genes and marker genes for DLPFC:Inh1 neurons. **Table S22**: Functional enrichment of RBFOX1 partners, also identified as rare variant associated genes and marker genes for DLPFC:Inh1 neurons.

## Data Availability

The datasets analyzed for the current study are available in Synapse. Bulk RNAseq data and snRNAseq data are accessible under the accession codes from their original publications syn8456629 and syn52293442, respectively, under controlled use conditions due to human privacy regulations. The multiplex immunofluorescence data, along with the results for all analyses, have been deposited on Synapse under the Synapse ID *syn63686123*. The code used for this publication is available at https://github.com/hidelab/ADresilience_CastanhoNaderi.

## Abbreviations

AD: Alzheimer’s disease
AD-PRS: Alzheimer’s disease polygenic risk scores
adj-P: Adjusted *P*-value
ADvsCTRL: Alzheimer’s disease versus control
ADvsPRE: Alzheimer’s disease versus presymptomatic
ADvsRES: Alzheimer’s disease versus resilient
ANGPT: Angiopoietin
ANGPT2: Angiopoietin-2
APOE: Apolipoprotein E
APP: Amyloid precursor protein
ATP8B1: ATPase phospholipid transporting 8B1
Aβ: Amyloid beta
BDNF: Brain-derived neurotrophic factor
BMP: Bone morphogenetic protein
BTC: Betacellulin
CA1: Cornu Ammonis 1
cGAS: Cyclic GMP-AMP synthase
CTRL: Control
DE: Differential expression
DEGs: Differentially expressed genes
DLPFC: Dorsolateral prefrontal cortex
EC: Entorhinal cortex
EC:Exc2: Excitatory neurons in the entorhinal cortex subtype 2
EC:Exc3: Excitatory neurons in the entorhinal cortex subtype 3
EGF: Epidermal growth factor
EGFR: Epidermal growth factor receptor
ERBB4: Erb-B2 receptor tyrosine kinase 4
ERK: Extracellular signal-regulated protein kinase
EWCE: Expression Weighted Celltype Enrichment
FC: Fold change
FDR: False discovery rate
FFPE: Formalin-fixed paraffin-embedded
GABA: Gamma-aminobutyric acid
GFAP: Glial fibrillary acidic protein
GO: Gene ontology
GWAS: Genome-wide association study
HC: Hippocampus
Hsp110: Heat shock protein 110
Hsp40: Heat shock protein 40
Hsp70: Heat shock protein 70
Hsp90: Heat shock protein 90
HSP90AA: Heat shock protein 90 alpha family class A
HSP90AA1: Heat shock protein 90 alpha family member A1
HSP90AB1: Heat shock protein 90 alpha family class B member 1
Hsps: Heat shock proteins
IFN-I: Interferon type I
IL-1β: Interleukin 1 beta
IL-6: Interleukin 6
ITGA5: Integrin alpha-5
ITGB1: Integrin beta-1
KIF26B: Kinesin family member 26B
KIT: KIT Proto-oncogene
KLF4: Kruppel-like factor 4
KLF4: Krüppel-like factor 4
L-R: Ligand-receptor
LCM: Laser capture microdissection
LINGO1: Leucine-rich repeat and Ig domain-containing protein 1
Log2FC: Log2 fold change
MAPK: Mitogen-activated protein kinase
MEF2A: Myocyte enhancer factor 2 A
MEF2C: Myocyte enhancer factor 2 C
mIF: Multiplex immunofluorescence
MSigDB: Molecular Signatures Database
ncWnt: Non-canonical Wnt
NGFR: Nerve growth factor receptor
NRN1: Neuretin
NRTK: Neurotrophin receptor tyrosine kinase
NT: Neurotrophin
NTRK2: Neurotrophic receptor tyrosine kinase 2
OPCs: Oligodendrocyte progenitor cells
PCxN: Pathway Co-expression Network
PI3K: Phosphoinositide 3-kinase
PLCγ: Phospholipase Cγ
PRE: Presymptomatic
PREvsCTRL: Presymptomatic versus control
PRS: Polygenic risk score
PS1: Presenilin 1
pTau: Phosphorylated tau
PVALB: Parvalbumin
RBFOX1: RNA Binding fox-1 Homolog 1
RELN: Reelin
RES: Resilient
RESvsCTRL: Resilient versus control
RESvsPRE: Resilient versus presymptomatic
RNAseq: RNA sequencing
ROSMAP: Religious Order Study and Memory and Aging Project
SNPs: Single nucleotide polymorphisms
snRNAseq: single-nucleus RNA sequencing
SORT1: Sortilin 1
SST: Somatostatin
SSTR1: Somatostatin receptor 1
SSTR2: Somatostatin receptor 2
TEK: TEK receptor tyrosine kinase
TGF-β: Transforming growth factor beta
TREM2: Triggering receptor expressed on myeloid cells 2
TULP: Tubby-like proteins
UMAP: Uniform Manifold Approximation and Projection
XBP1s: X-box binding protein 1 spliced
