# Supplementary material for: Molecular hallmarks of excitatory and inhibitory neuronal resilience to Alzheimer’s disease
Source: Mol Neurodegener. 2025 Oct 1;20:103. doi: 10.1186/s13024-025-00892-3 (PMC12487324; doi:10.1186/s13024-025-00892-3)
Supplement: Supplementary file 1 — Supplementary Figure 1 to 21 [file 13024_2025_892_MOESM1_ESM.pdf]

Molecular hallmarks of excitatory and inhibitory neuronal resilience to Alzheimer's disease

Isabel Castanho[1,2]*, Pourya Naderi Yeganeh[1,2]*, Carles A. Boix[3,4], Sarah L. Morgan[1,2,5], Hansruedi Mathys[6,7], Dmitry Prokopenko[1,8], Bartholomew White[1,2], Larisa M. Soto[1,2], Giulia Pegoraro[1,10], Saloni Shah[1], Athanasios Ploumakis[1,11], Nikolas Kalavros[1,2,4,11], David A. Bennett[12], Christoph Lange[13], Doo Yeon Kim[1,8,9], Lars Bertram[14,15], Li-Huei Tsai[4,7,16], Manolis Kellis[3,4], Rudolph E. Tanzi[1,8,9,^], Winston Hide[1,2,^]

Extended Results

Resilience versus preclinical AD

To further characterize resilience, we included a presymptomatic category (PRE) in our differential gene expression analysis, made up of individuals with mild cognitive impairment and advanced AD pathology (see **Methods**). A comparison of presymptomatic versus resilient subjects yielded no DEGs, whereas there were 68 DEGs between presymptomatic and AD (**Table S4**). These results indicate that the majority of the transcriptomic changes in the AD continuum emerge during the transition from a resilient/presymptomatic state to late-stage AD dementia, rather than at the onset of pathology without cognitive impairment. The major difference between the AD group with the presymptomatic and resilience groups was the presence/absence of dementia.

A comparison of AD with control and presymptomatic groups identified 51 dysregulated pathways (q-value < 0.1) in ADvsCTRL (**Table S8**). Concordant with gene-level analysis, no significant changes were identified between resilience and presymptomatic groups (**Table S9**). We also found 290 dysregulated pathways (q-value < 0.1) in ADvsPRE (**Table S10**). Taken

together, pathway activity analysis defines resilience and presymptomatic states with maintenance of RNA/DNA processing pathways lost during transition to AD dementia, and replacement by activation of signaling cascades.

Functional characterization of resilience transcriptomes into pathways

To functionally characterize differentially regulated pathways in resilience and AD, we applied pathway-activity analysis (**Methods**) to summarize the overall activity of genes within known pathways. Applied to ROSMAP bulk RNAseq data and using 1329 background pathways from the MSigDB database, we identified significant dysregulation of 99 pathways (q-value < 0.1) in ADvsRES (**Table S6**; 6 pathways with q-value < 0.05). Leveraging unsupervised clustering and an independent canonical co-expression map of the pathways, we identified two super-groups of pathways that dominated the differences between AD and resilience subjects (**Figure S2B, Table S7**). The largest cluster included upregulation of 43 signaling pathways in AD, including NFKB (nuclear factor kappa-light-chain-enhancer) activation, MAPK (mitogen-activated protein kinases) activation, and FAK (focal adhesion kinase) signaling. The second largest cluster included 19 downregulated pathways associated with RNA machinery and metabolism in AD compared to resilience, including RNA degradation and RNA metabolism, calcium signaling, and protein metabolism and export.

GFAP

Mirroring the bulk RNAseq findings, the single-cell expression of *GFAP* in the DLPFC was significantly upregulated in resilience (**Figure S9** and **Table S13**) relative to both AD (ADvsRES) and controls (RESvsCTRL; top DEG in astrocytes). This parallel upregulation at the tissue and cellular levels reinforces GFAP as a hallmark of an intermediate, resilient state.

APOE

The expression levels of *APOE* (coding for apolipoprotein E), a well-known gene in the context of AD, showed an increase in AD in microglia in all brain regions, but not in resilient individuals (**Figure S9** and **Table S13**). In AD astrocytes, despite being upregulated in the DLPFC, *APOE* was downregulated in the EC in AD and in resilient subjects. APOE, particularly the ε4 variant, is closely associated with microglial function and neuroinflammation [1]. It is a ligand for TREM2, which regulates microglial activation and the transition to a phagocytic phenotype [2]. Besides microglia, APOE also affects astrocytic function. For example, the ε4 variant of APOE has been linked to less efficient lipid transport and cholesterol homeostasis in astrocytes, contributing to the overall neuroinflammatory environment [3]. Taken together, our results point to microglial changes that align with a suppression of neuroinflammatory responses in cognitive resilience, as previously reported in the literature. Several studies have demonstrated that a suppressed neuroinflammatory response plays a key role in cognitive resilience to AD [4–6]. Resilient individuals have been shown to have reduced expression of inflammatory markers, along with increased levels of cytokines involved in inflammation resolution, such as IL-1β and IL-6 [4].

PLCG2

PLCG2, previously implicated by the protective gain-of-function variant P522R [7], was robustly up-regulated in every major cell class in AD EC and HC (**Table S13**), mirroring pan-microglial activation seen at late disease stages. In resilient brains, however, *PLCG2* was significantly increased only in excitatory neurons, while being suppressed in EC astrocytes (**Table S13**), suggesting a more nuanced, cell-restricted deployment of *PLCG2* signaling that may preserve debris-clearance capacity while dampening glia-driven inflammation. It is worth noting that the P522R allele enhances enzyme activity [8], whereas our data capture transcriptional regulation; the two mechanisms are not mutually exclusive: resilient brains may combine moderate,

compartmentalized *PLCG2* expression with intrinsically higher catalytic efficiency, whereas late-stage AD appears to compensate through broad transcriptional upregulation that coincides with detrimental neuroinflammation.

Markers of resistance and protection-associated inhibitory neurons

Among the protective genes highly expressed in SST+ DLPFC:Inh1 cells, we further investigated *RBFOX1* (RNA binding fox-1 homolog 1) and *KIF26B* (kinesin family member 26B), selected based on high neuronal expression (*Human Protein Atlas)* and availability of suitable antibodies for targeting. Multiplex immunofluorescence (mIF) imaging for SST, GABA, RBFOX1, KIF26B, and Aβ in an independent cohort of formalin-fixed paraffin-embedded (FFPE) human DLPFC sections (see **Methods**) (**Figure S13** and **Table S19**) confirmed co-expression of RBFOX1 and KIF26B in SST+ cortical neurons.

We identified and annotated cellular populations based upon clustering of the single-cell mIF data (see **Methods**) (**Figure S13B**) [9,10]. Pearson correlation analysis confirmed cellular co-expression of defining markers, establishing the potential value of rare-variant associated genes as co-factors of somatostatin expression (**Figure S13D**). The overall proportion of SST+ cells varied between AD, resilient, and control groups. Despite showing the expected decrease in AD (**Figure 13F**), a Dirichlet test failed to reach statistical significance, which may be explained by the small effect size and the low number of donors tested in this cohort (n = 4-6 per group). Nonetheless, we did observe a significant decrease in the cell-mean intensity of SST in SST+ neurons from AD and resilient subjects as compared to controls (**Figure 13F**). A subpopulation of SST+ neurons, which expressed intracellular amyloid-beta (Aβ+) showed a significant increase in proportion in AD versus CTRL (**Figure S13H**). No differences in cell proportions were detected for a specific subpopulation that expressed all selected markers (SST+ RBFOX1+ KIF26B+ GABA+ cluster) at the protein level (**Figure 13G**).

Supplementary figures

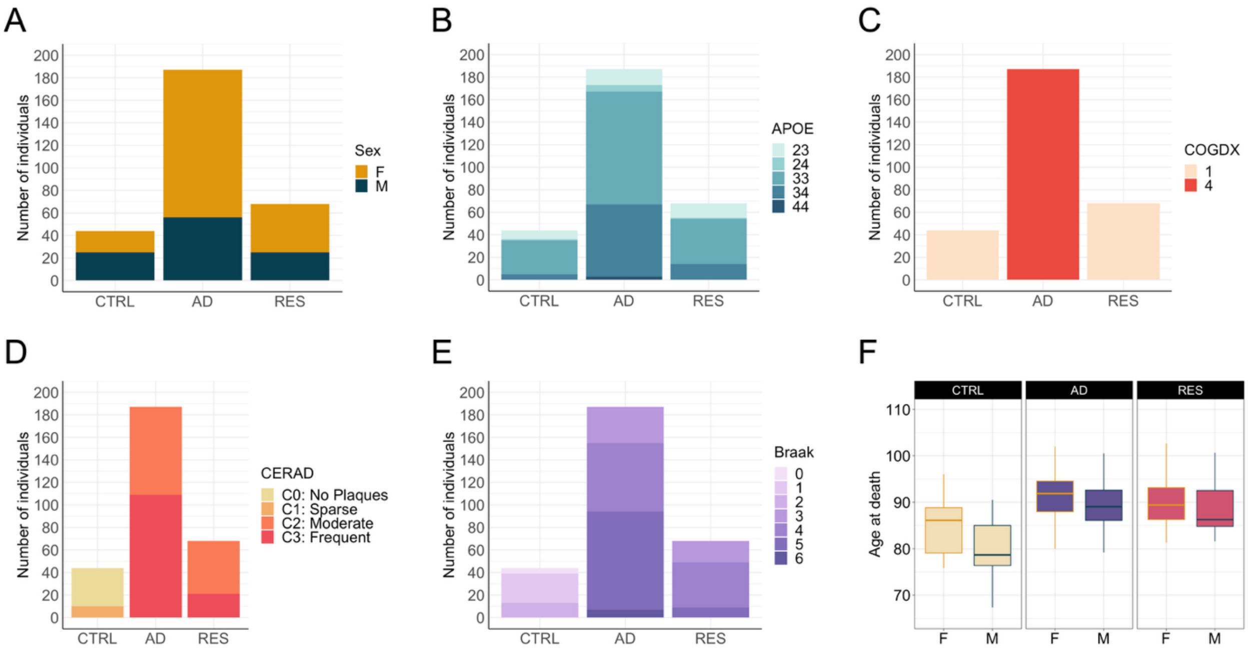

Figure S1. Characteristics of bulk RNAseq ROSMAP subjects used in this study (DLPFC).
(A) Sex distribution.
(B) *APOE* genotype distribution.
(C) Clinical consensus diagnosis of cognitive status at the time of death (final consensus cognitive diagnosis, "cogdx" variable from RUSH Alzheimer's Disease Center (RADC) Research Resource Sharing Hub).
(D) Consortium to Establish a Registry for Alzheimer's Disease (CERAD) score.
(E) Braak stage distribution.
(F) Age distribution, by sex. The central horizontal line of the box plots depicts the median, and the lower and upper hinges correspond to the first and third quartiles (the 25th and 75th percentiles).
CTRL: Control; AD: Alzheimer's disease; RES: Resilient. F: females; M: males.

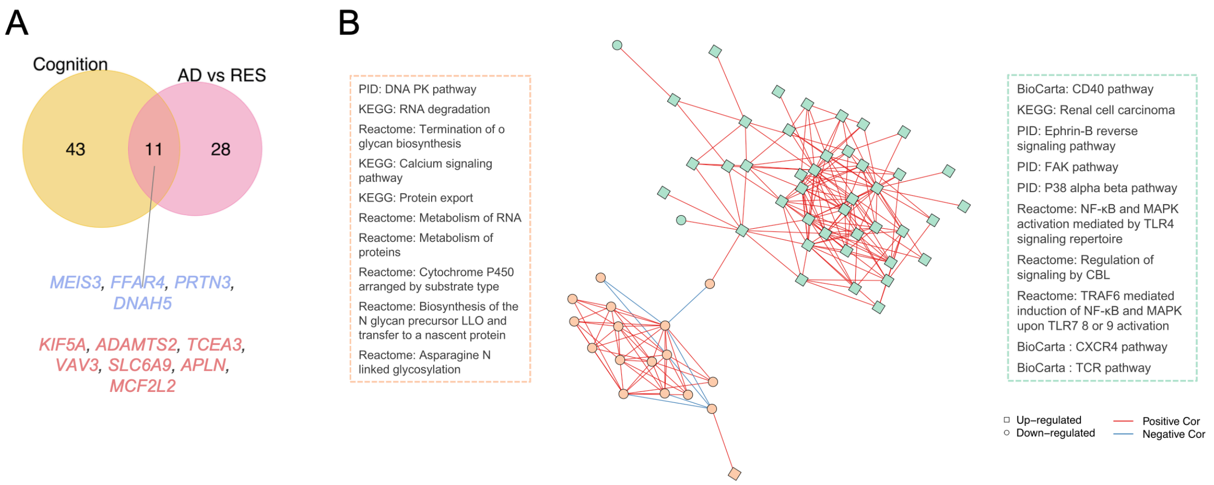

Figure S2. Pathway signatures of cognitive resilience against AD pathology.
(A) Venn diagram showing common genes identified as DEGs in ADvsRES and identified as associated with cognitive decline. Cognitive-associated genes were identified using ordinal categorical regression (proportional odds model), irrespective of group classification, and adjusted for the burden of pathology. Down-regulated genes are shown in blue, and upregulated genes in red (adj-P < 0.1; **Table S5**).
(B) Two major classes of dysregulated functions in ADvsRES identified by pathway activity analysis. Nodes represent pathways with significant dysregulation of activity (q-value < 0.1) in ADvsRES. Out of a total of 99 dysregulated pathways between AD and RES, 62 were organized into two unsupervised clusters of expression. Node shapes denote upregulation and downregulation in AD. Edges represent co-expression of pathways based on the Pathway Co-expression Network background. Pathway activity profiles were determined using the PanomiR software package. Pathway dysregulation p-values were determined using the Limma package's linear regression models contrasting between ADvsRES and accounting for confounding covariates such as age, batch, and RNA integrity number. Related to **Tables S6-S7**.

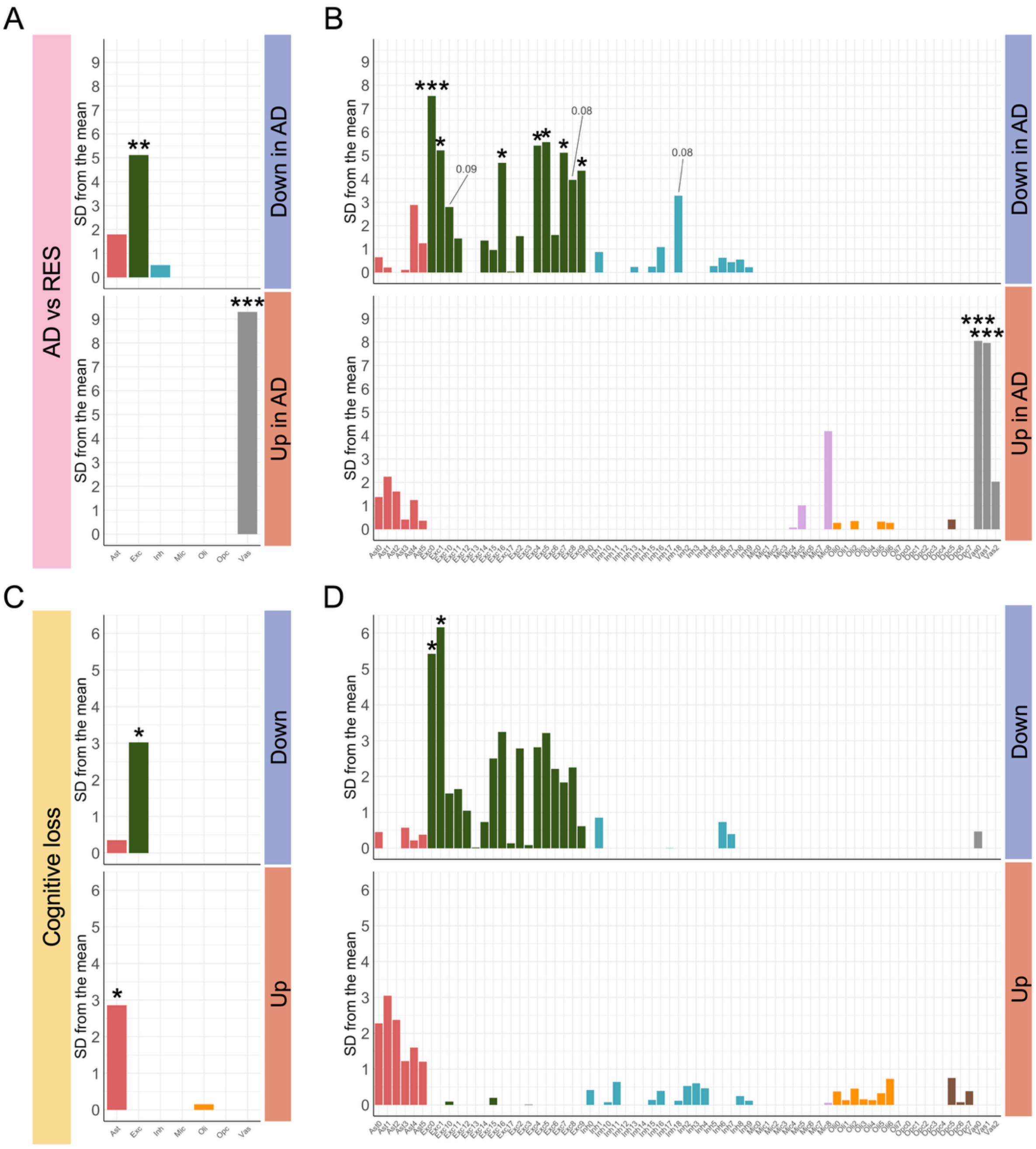

Figure S3. Expression weighted cellular enrichment results for differentially expressed genes in bulk RNAseq data from DLPFC.

(A-B) Cellular enrichment (*EWCE*) of genes uniquely differentially expressed in ADvsRES (61 DEGs) in major cell types (A) and cell subtypes (B) identified in the DLPFC.

(C-D) Cellular enrichment (*EWCE*) for genes associated with cognitive decline in major cell types (C) and cell subtypes (D). Down (top): genes downregulated with cognitive decline; Up (bottom): genes upregulated with cognitive decline.

The Y-axis represents the number of standard deviations from the bootstrap mean, corresponding to the enrichment of the specified cell type in the gene list investigated. Stars represent Bonferroni-adjusted P-values: * adj-P < 0.05, ** adj-P < 0.01, *** adj-P < 0.001.

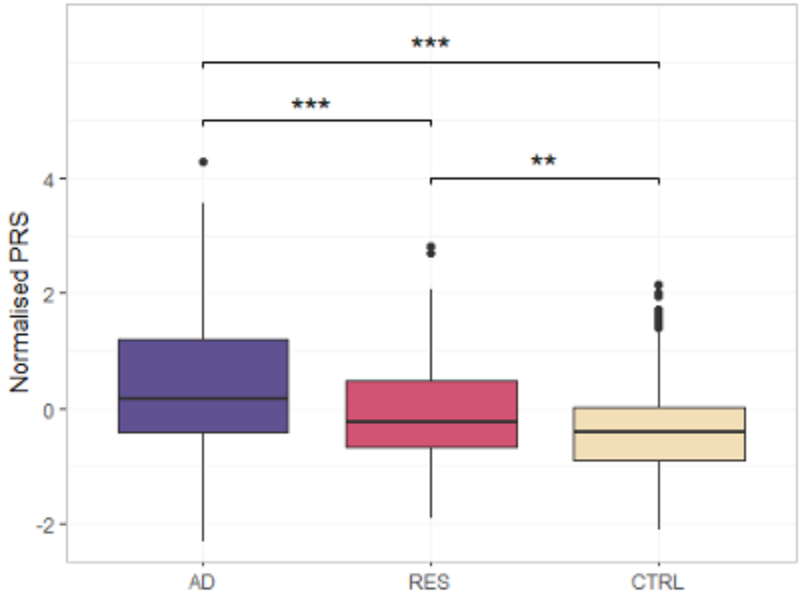

Figure S4. Sample classification details and cognitive resilience relative to AD polygenic risk.
(A) AD polygenic risk scores (AD-PRS) were calculated for each subject from ROSMAP with genetic data available. The central horizontal line of the box plots depicts the median, and the lower and upper hinges correspond to the first and third quartiles (the 25th and 75th percentiles). The circles represent outliers, and stars denote Bonferroni-adjusted P-values.
** adj-P < 0.01, *** adj-P < 0.001.
CTRL: Control, AD: Alzheimer's disease, RES: Resilient.

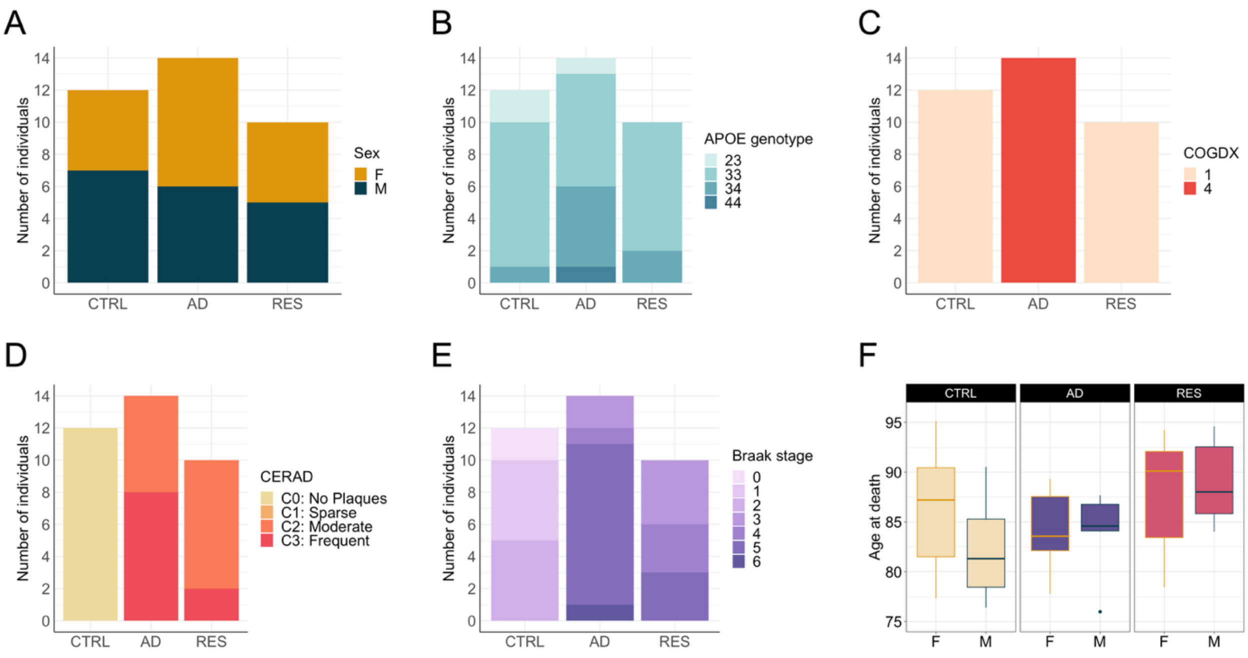

Figure S5. Characteristics of ROSMAP subjects with snRNAseq from the dorsolateral prefrontal cortex (DLPFC) used in this study.
(A) Sex distribution.
(B) APOE genotype distribution.
(C) Clinical consensus diagnosis of cognitive status at time of death (final consensus cognitive diagnosis, “cogdx” variable from RUSH Alzheimer's Disease Center (RADC) Research Resource Sharing Hub).
(D) Consortium to Establish a Registry for Alzheimer's Disease (CERAD) score.
(**E)** Braak stage distribution.
(F) Age distribution, by sex. The central horizontal line of the box plots depicts the median, and the lower and upper hinges correspond to the first and third quartiles (the 25th and 75th percentiles). Circles represent outliers.
CTRL: Control, AD: Alzheimer’s disease, RES: Resilient. F: females, M: males.

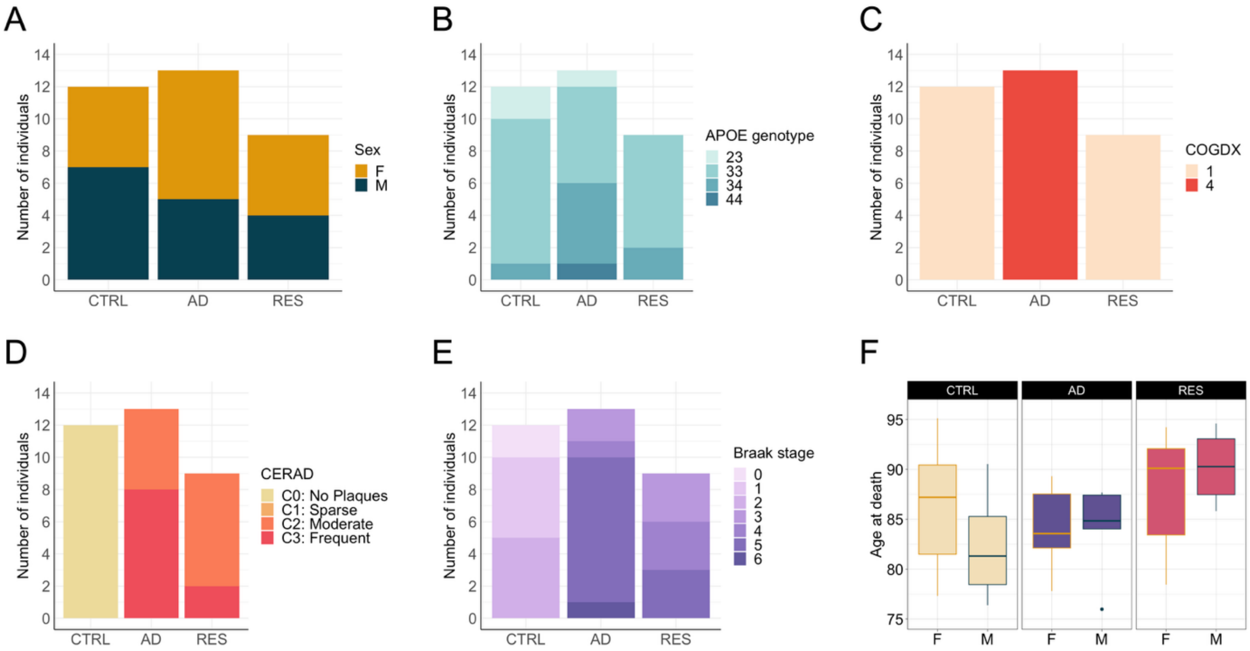

Figure S6. Characteristics of ROSMAP subjects with snRNAseq from the entorhinal cortex (EC) used in this study.
(A) Sex distribution.
(B) APOE genotype distribution.
(C) Clinical consensus diagnosis of cognitive status at time of death (final consensus cognitive diagnosis, “cogdx” variable from RUSH Alzheimer's Disease Center (RADC) Research Resource Sharing Hub).
(D) Consortium to Establish a Registry for Alzheimer's Disease (CERAD) score.
(E) Braak stage distribution.
(F) Age distribution, by sex. The central horizontal line of the box plots depicts the median, and the lower and upper hinges correspond to the first and third quartiles (the 25th and 75th percentiles). Circles represent outliers.
CTRL: Control, AD: Alzheimer’s disease, RES: Resilient. F: females, M: males.

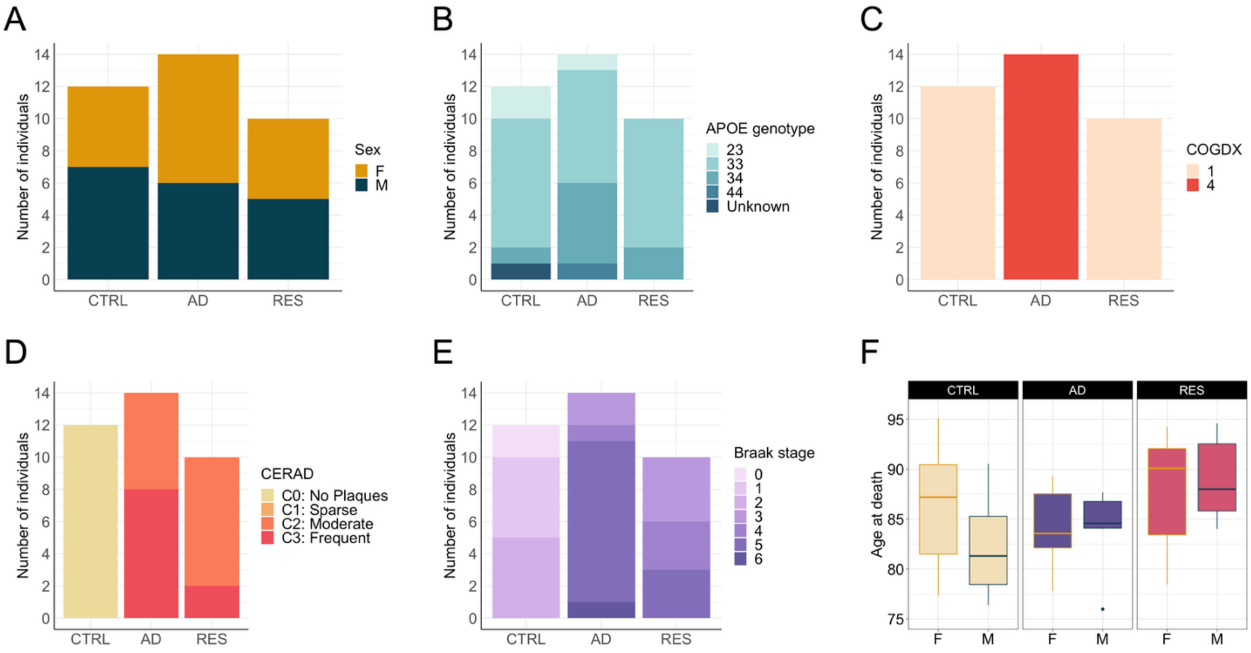

Figure S7. Characteristics of ROSMAP subjects with snRNAseq from the hippocampus (HC) used in this study.
(A) Sex distribution.
(B) APOE genotype distribution.
(C) Clinical consensus diagnosis of cognitive status at time of death (final consensus cognitive diagnosis, “cogdx” variable from RUSH Alzheimer's Disease Center (RADC) Research Resource Sharing Hub).
(D) Consortium to Establish a Registry for Alzheimer's Disease (CERAD) score.
(E) Braak stage distribution.
(F) Age distribution, by sex. The central horizontal line of the box plots depicts the median, and the lower and upper hinges correspond to the first and third quartiles (the 25th and 75th percentiles). Circles represent outliers.
CTRL: Control, AD: Alzheimer’s disease, RES: Resilient. F: females, M: males.

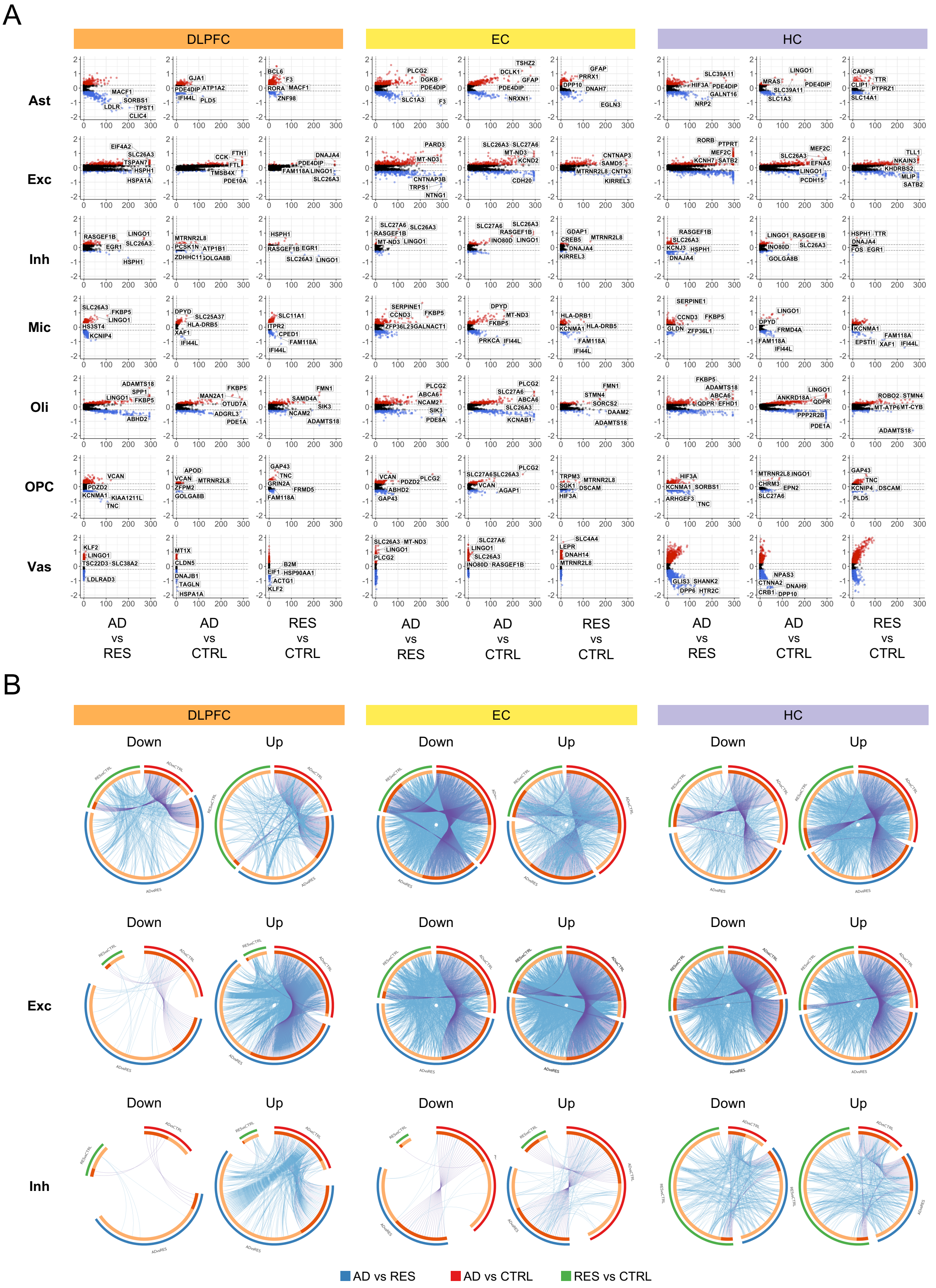
A
DLPFC
EC
HC
Ast
Exc
Inh
Mic
Oli
OPC
Vas
AD vs RES
AD vs CTRL
RES vs CTRL
B
Down
Up
AD vs RES
AD vs CTRL
RES vs CTRL

Figure S8. Cell-specific transcriptomic changes in AD resilience.
(A) Volcano plots showing significantly (adj-P < 0.1) differentially expressed genes (DEGs) in each brain region tested (DLPFC, EC, and HC) in ADvsRES, ADvsCTRL, and RESvsCTRL. DEGs with log2FC < -0.2 are highlighted in blue, and DEGs with log2FC > 0.2 are highlighted in red. The horizontal lines represent Bonferroni-adjusted P (adj-P) = 0.1.
(B) Circular plots showing overlapping genes and ontologies from each comparison for excitatory and inhibitory neurons from each brain region investigated. Each outer arc represents the identity of each gene list (blue: ADvsRES, red: ADvsCTRL, green: RESvsCTRL). Each inner arc shows the genes that are shared by multiple lists in dark orange and genes that are unique to that gene list in light orange. Purple lines connect the same gene, shared by multiple lists. Blue lines represent genes that fall under the same ontology term. Gene ontology enrichment analysis was performed using Metascape.
CTRL: Control, AD: Alzheimer’s disease, RES: Resilient.
Ast: Astrocytes, Exc: Excitatory neurons, Inh: Inhibitory neurons, Mic & Imm: Microglia and immune cells, Oli: Oligodendrocytes, OPC: Oligodendrocyte progenitor cells, Vas & Epi: Vascular and epithelial cells.
DLPFC: Dorsolateral prefrontal cortex, EC: Entorhinal cortex, HC: Hippocampus.

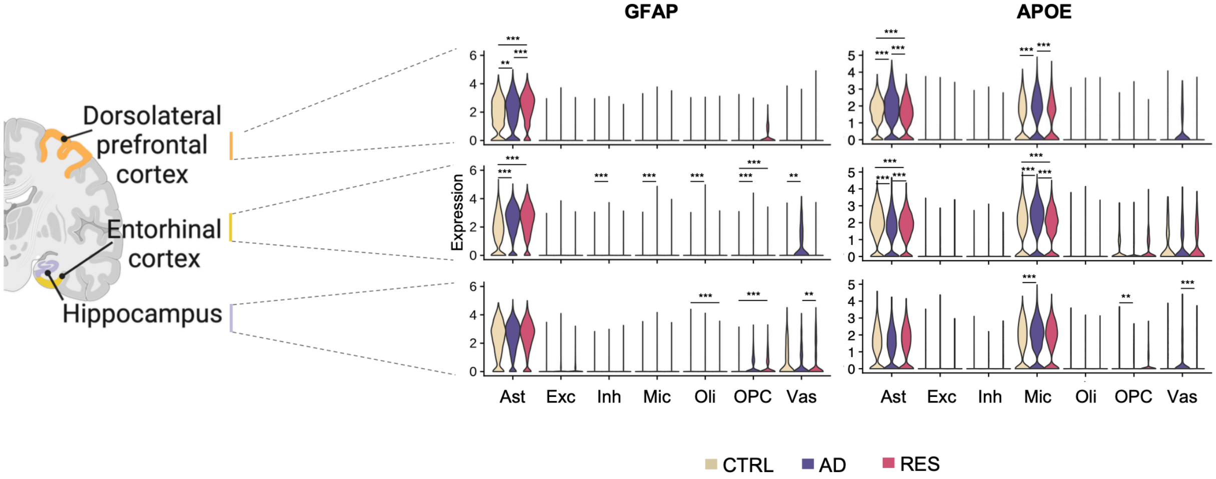

Figure S9. Changes in the expression of *GFAP*.
Violin plots showing gene expression for *GFAP* across different major cell types from each brain region. Log2FC, adjusted P-values (adj-P), and direction of change (first diagnostic group compared to the second group) are shown in Table S13. Differential expression performed using MAST in *Seurat*. Stars reflect Bonferroni-adjusted P-values (**Table S13**).
* adj-P < 0.05, ** adj-P < 0.01, *** adj-P < 0.001. Sample size distributions are shown in **Table S11**.
Figure created in part with BioRender.com.

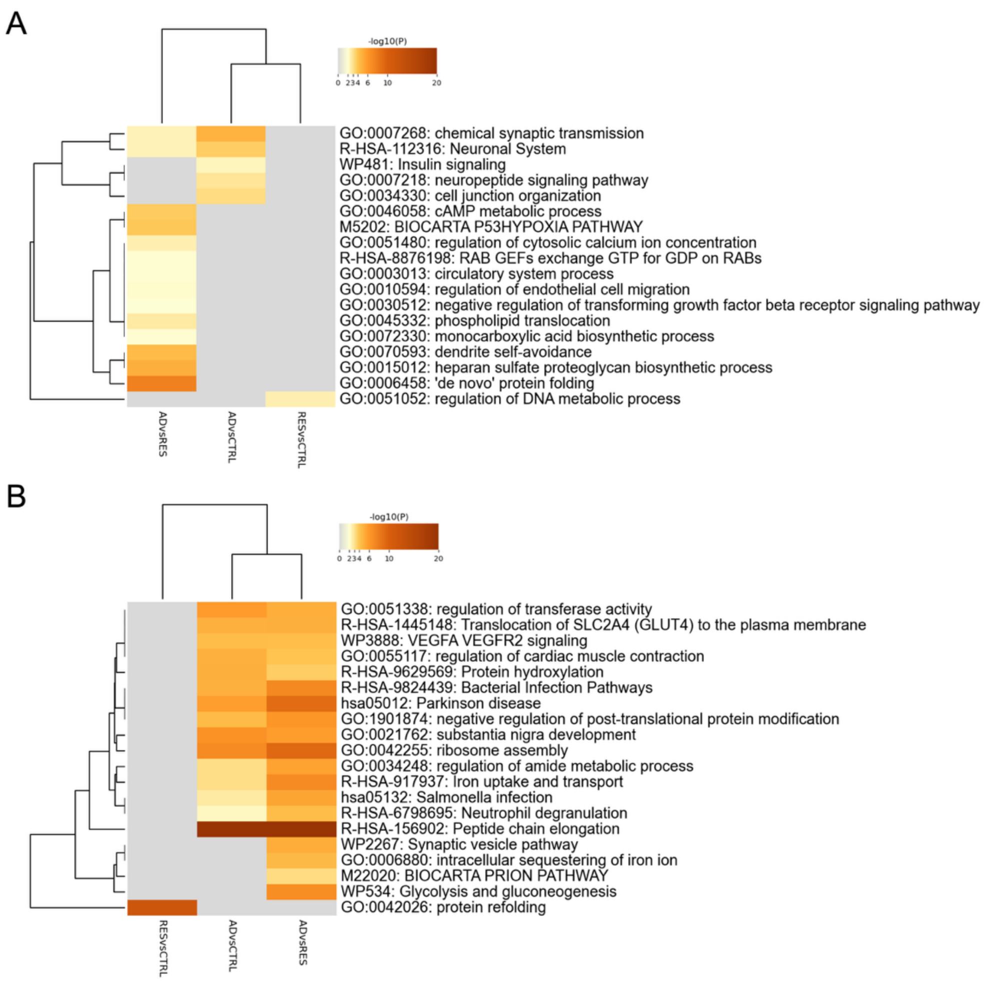

Figure S10. Significant terms from gene ontology enrichment analysis for DEGs identified in excitatory neurons from the DLPFC.

(A-B) Heatmaps showing enriched gene ontology clusters across gene lists from each of the three comparisons (ADvsRES, ADvsCTRL, RESvsCTRL). The cells in each heatmap are colored by their respective p-values, with gray cells indicating a lack of enrichment for that term in the corresponding gene list. The terms with the best p-values within each cluster are displayed in the dendrogram. Cumulative hypergeometric p-values and enrichment factors were calculated and used for filtering. Significant terms were hierarchically clustered into a tree based on Kappa-statistical similarities among their gene memberships, with 0.3 kappa score applied as the threshold to cast the tree into clusters.

(A) Genes downregulated in the first group compared to the second group for each comparison (adj-P < 0.1; log2FC < -0.2).

(B) Genes upregulated in the first group compared to the second group for each comparison (adj-P < 0.1; log2FC > 0.2).

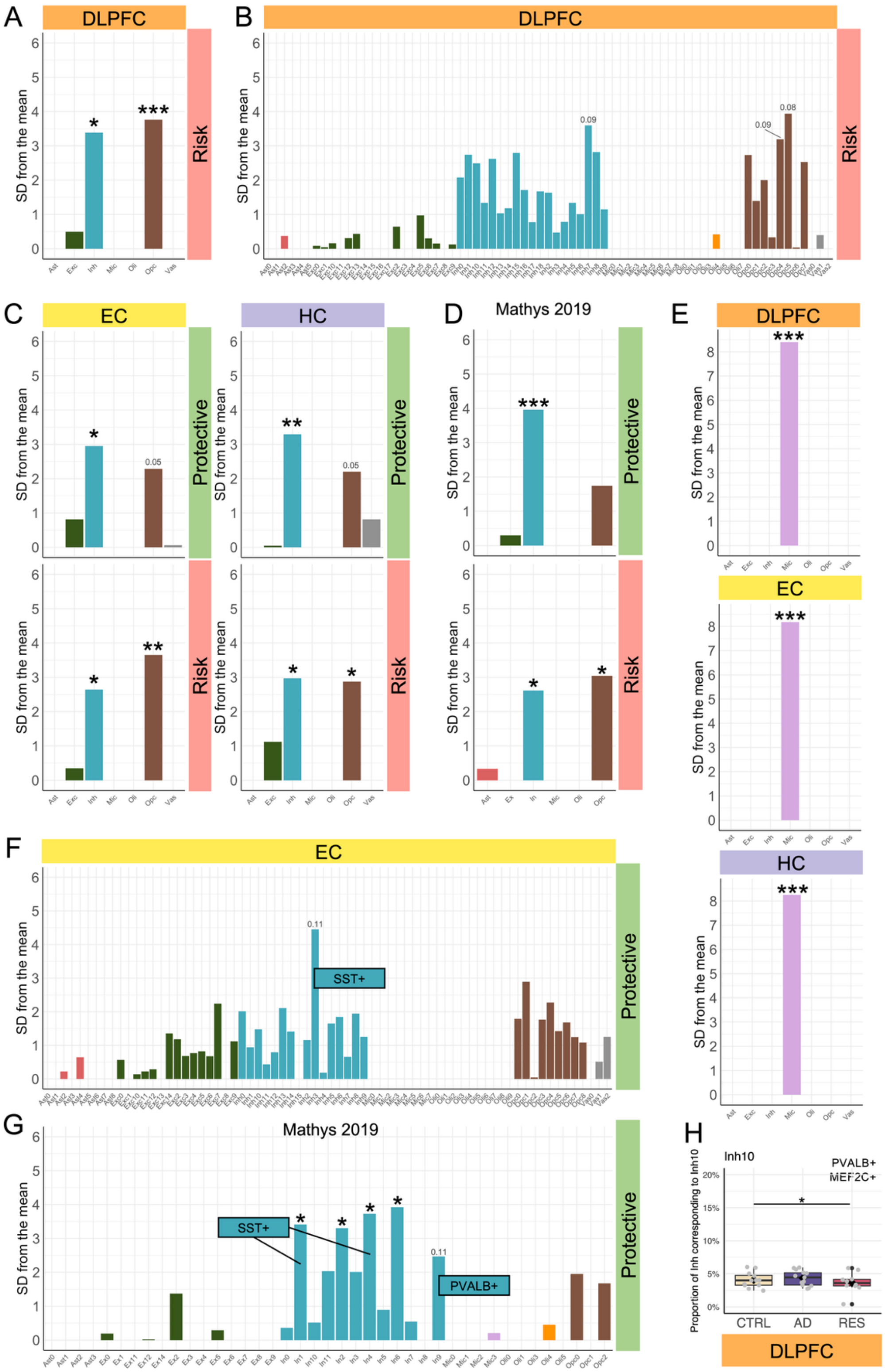

Figure S11. Expression-weighted cellular enrichment results for genes identified from rare variants.

(A) Cellular enrichment of genes identified from risk genetic rare variants in the DLPFC for major cell types.
(B) Cellular enrichment of genes identified from risk genetic rare variants in the DLPFC for cell subtypes.
(C) Cellular enrichment of genes identified from protective and risk genetic rare variants in the EC and HC for major cell types.
(D) Cellular enrichment of genes identified from protective and risk genetic rare variants in the Mathys et al. 2019 dataset (DLPC) for major cell types.
(E) Genes identified from common variants from Bellenguez et al. 2022 in major cell types.
(F) Cellular enrichment of genes identified from protective rare variants in the EC for cell subtypes.
(G) Cellular enrichment of genes identified from protective rare variants in the Mathys et al. 2019 dataset (DLPC) for cell subtypes.
(E) Cell proportion changes for PVALB+ DLPFC:Inh10.
Stars represent Bonferroni-adjusted P-values: * adj-P < 0.05, ** adj-P < 0.01, *** adj-P < 0.001
CTRL: Control, AD: Alzheimer’s disease, RES: Resilient.
DLPFC: Dorsolateral prefrontal cortex, EC: Entorhinal cortex, HC: Hippocampus.

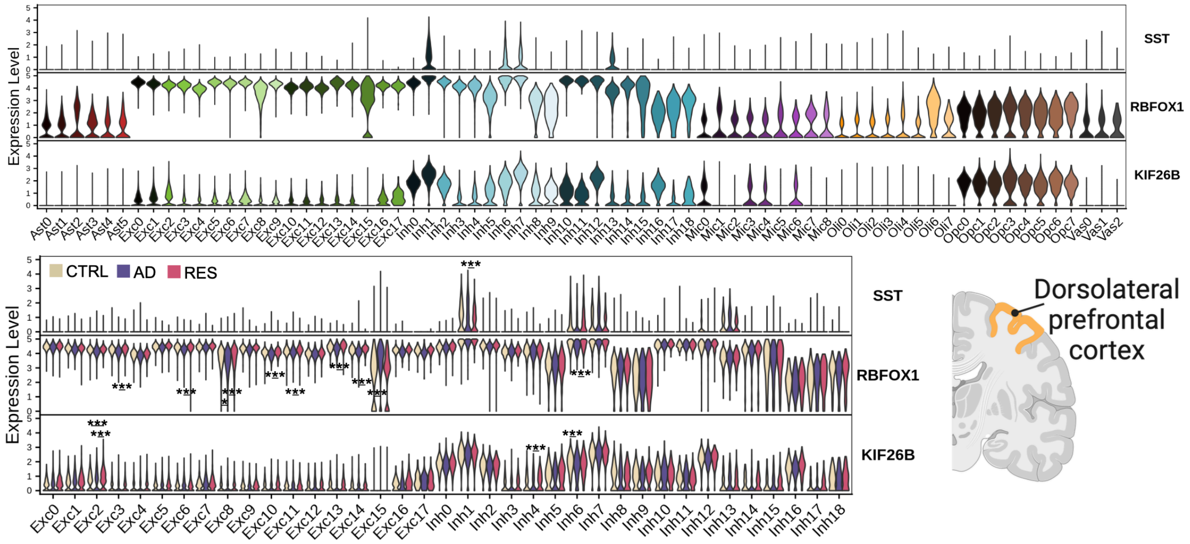

Figure S12. Gene expression distributions for SST, RBFOX1, and KIF26B.
Violin plots showing gene expression levels of SST, RBFOX1, and KIF26B for each subtype (top) and neuronal subtypes by diagnostic group (bottom) in the DLPFC. Stars represent Bonferroni-adjusted P-values: * Adj-P < 0.05, *** Adj-P < 0.001.
CTRL: Control, AD: Alzheimer’s disease, RES: Resilient.
DLPFC: Dorsolateral prefrontal cortex.
Figure created in part with BioRender.com.

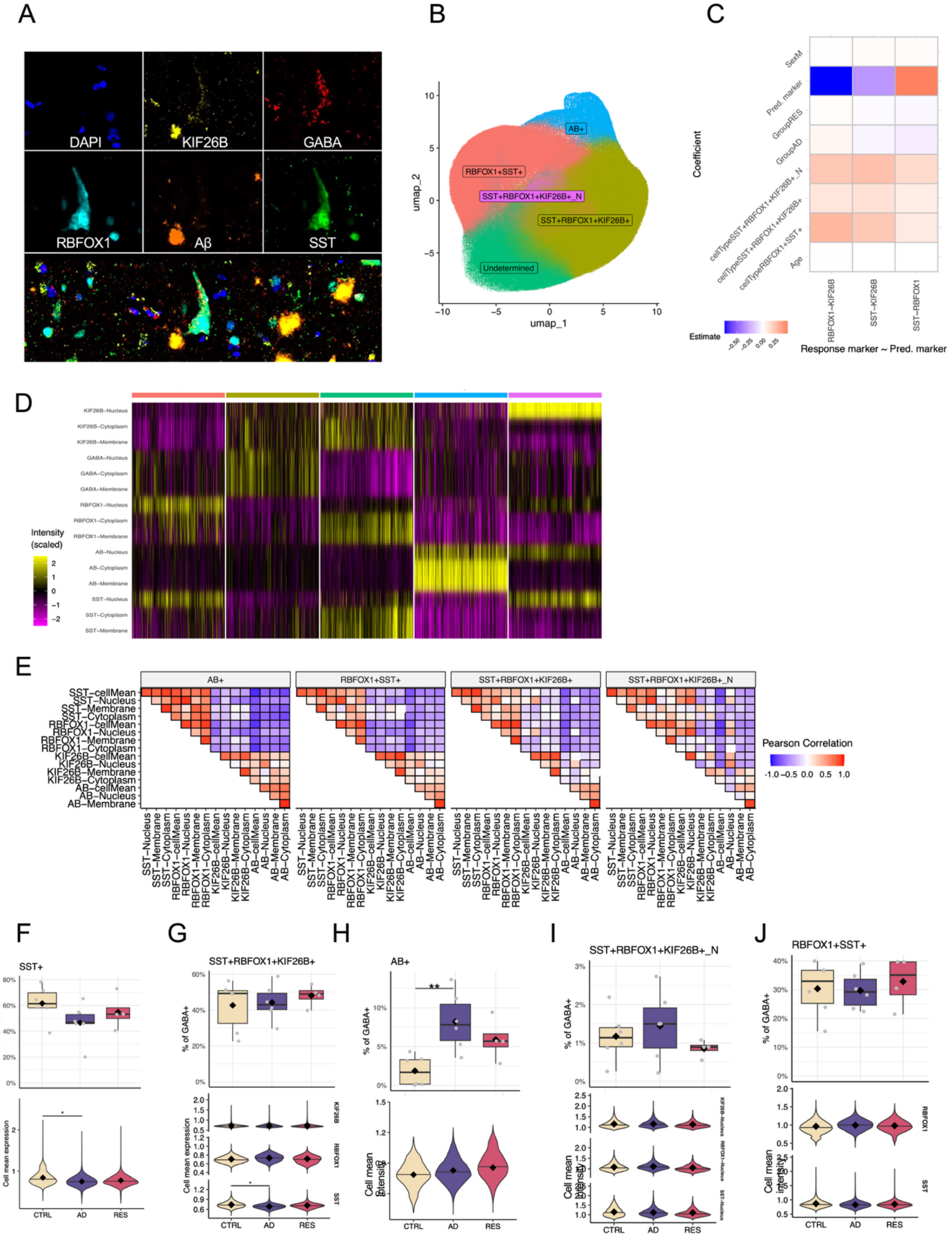

Figure S13. Protein expression assessed by multiplex immunostaining (mIF) for SST, RBFOX1, and KIF26B.

Protein staining of SST+ neurons in an independent cohort. Sample size distributions shown in **Table S18**.
(A) Representative image from mIF staining of markers in a resilient DLPFC brain section.
(B) UMAP showing the populations identified in the DLPFC in the BIDMC cohort from multiplex immunofluorescence (mIF) staining for GABA, SST, RBFOX1, KIF26B, and Aβ. $N_{subjects}$ = 16 (6 CTRL, 6 AD, 4 RES), N_{cells} = 1310803 (CTRL = 465007, AD = 559794, RES = 286002 cells).
(C) Coefficients of predictors used in linear models of marker intensities.
(D) Heatmap of normalized intensity of protein markers in a random subset of 500 cells from each cell type (indicated by the top color bar). Cell type labels shown in (B).
(E) Correlation of inhibitory neuronal targets normalized intensity across identified cell subpopulations. Each square is colored by the Pearson correlation coefficient corresponding to the pair of markers indicated in the x and y axes. Solid black squares indicate significant correlations at an FDR of 0.05.
(F) Proportions of all SST+ (GABA+) cells in each subject across diagnostic groups (top). Distribution of mean SST normalized intensities in SST+ cells (bottom). $N_{subjects}$ = 16 (6 CTRL, 6 AD, 4 RES), N_{cells} = 1,279,938 (CTRL = 434,270, AD = 559,681, RES = 285,987).
(G) Distribution of proportions of all SST+ RBFOX1+ KIF26B+ (GABA+) cells (top). Distribution of mean intensities of each marker in SST+ RBFOX1+ KIF26B+ cells (bottom). $N_{subjects}$ = 16 (6 CTRL, 6 AD, 4 RES), N_{cells} = 721,126 (CTRL = 261,938, AD = 315,542, RES = 143,646).
(H-I) Box plots (top) showing the results for differential cell proportions of additional clusters shown in (B) and violin plots (bottom) showing the distribution of their corresponding cell-level protein intensities (normalized immunofluorescence levels) in each population. Diamonds show the grand mean of subject-level mean normalized intensities across cells.
Stars in top (F-J) indicate FDR-adjusted P-values from a Dirichlet multinomial regression. Stars in bottom (F-J) indicate nominal P-values of a Wilcoxon test on the subject-level means; diamonds show the grand mean.
CTRL: Control, AD: Alzheimer's disease, RES: Resilient.
DLPFC: Dorsolateral prefrontal cortex.
P-values in F-J were adjusted for multiple testing using the FDR method: * adj-P < 0.05, ** adj-P < 0.01.

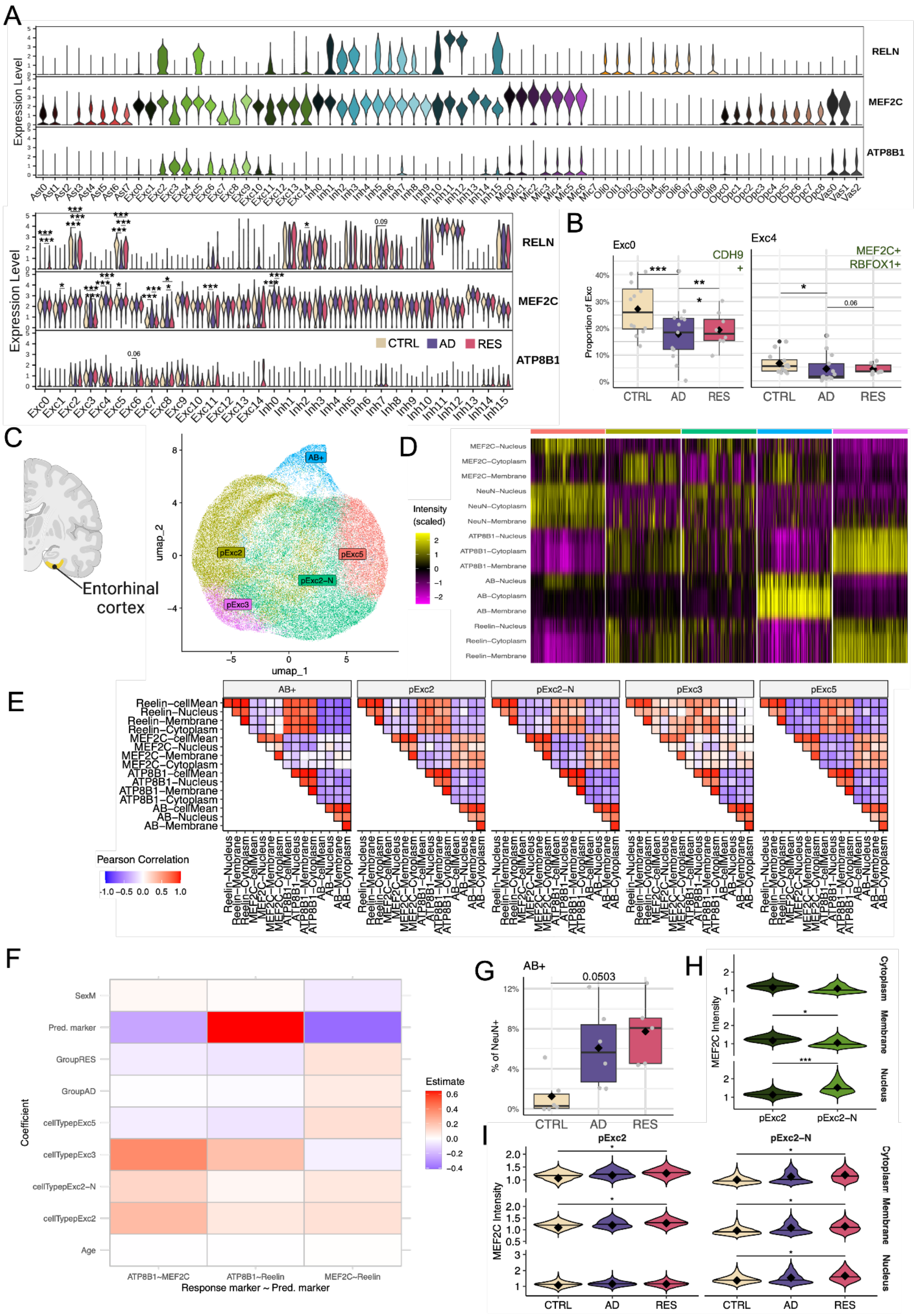
A
RELN
MEF2C
ATP8B1
Expression Level
B
Exc0
Exc4
CDH9
MEF2C+
RBFOX1+
Proportion of Exc
CTRL
AD
RES
C
Entorhinal cortex
umap_1
umap_2
AB+
pExc2
pExc5
pExc2−N
pExc3
D
Intensity (scaled)
E
Pearson Correlation
F
Coefficient
Estimate
Response marker ~ Pred. marker
G
AB+
% of NeuN+
H
MEF2C Intensity
I
pExc2
pExc2−N
Cytoplasm
Membrane
Nucleus

Figure S14. Gene expression profiles for RELN, MEF2C, and ATP8B1, and protein expression assessed by multiplex immunostaining (mIF) in excitatory neurons associated with resilience.

(A) Violin plots showing gene expression levels (snRNAseq) for RELN, MEF2C, and ATP8B1 for all subtypes (top) and neuronal subtypes by group (bottom) in the EC (related to Figure 4). Cell proportion changes in additional vulnerable excitatory subtypes from the entorhinal cortex, as reported by Leng et al., 2021. Stars represent Bonferroni-adjusted P-values.

(B) Cell proportion changes for CDH9+ EC:Exc0 cells (left) and cell proportion changes for RBFOX1+ $MEF2C_{high}$ EC:Exc4 (right). Stars represent FDR-adjusted P-values.

(C) UMAP showing the populations identified in the EC in the BIDMC cohort from multiplex immunofluorescence (mIF) staining for NeuN, MEF2C, ATP8B1, RELN, and Aβ. $N_{subjects}$ = 16 (6 CTRL, 6 AD, 5 RES), N_{cells} = 81549 cells.

(D) Heatmap of normalized intensity of protein markers in a random subset of 500 cells from each cell type (indicated by the top color bar). Cell type labels shown in (B).

(E) Correlation of inhibitory neuronal targets normalized intensity across identified cell subpopulations. Each square is colored by the Pearson correlation coefficient corresponding to the pair of markers indicated in the x and y axes. Solid black squares indicate significant correlations at an FDR of 0.05.

(F) Coefficients of predictors used in linear models of marker intensities.

(G) Box plots showing cell proportion changes in the cluster AB+ shown in (B), corresponding to neurons expressing high protein levels of Aβ.

(H) Violin plots showing protein expression (normalized immunofluorescence intensity levels) of MEF2C in different compartments (cytoplasm, membrane, and nucleus) for the clusters pExc2 and pExc2-N ($MEF2C_{high}$ ATP8B1+ RELN+) shown in (D).

(I) Violin plots showing distributions of protein expression (normalized immunofluorescence intensity levels) of MEF2C in different compartments (cytoplasm, membrane, and nucleus) for the clusters pExc2 and pExc2-N ($MEF2C_{high}$ ATP8B1+ RELN+) by diagnostic group (CTRL, AD, RES). In (H) and (I) stars indicate significance level based on nominal P-values of a Wilcoxon test performed on the subject-level means. Diamonds show the grand mean of subject-level mean normalized intensities across cells.

Stars in **G-H** represent FDR-adjusted P-values.

* adj-P < 0.05, ** adj-P < 0.01, *** adj-P < 0.001.

CTRL: Control, AD: Alzheimer's disease, RES: Resilient.

EC: Entorhinal cortex.

Figure created in part with BioRender.com.

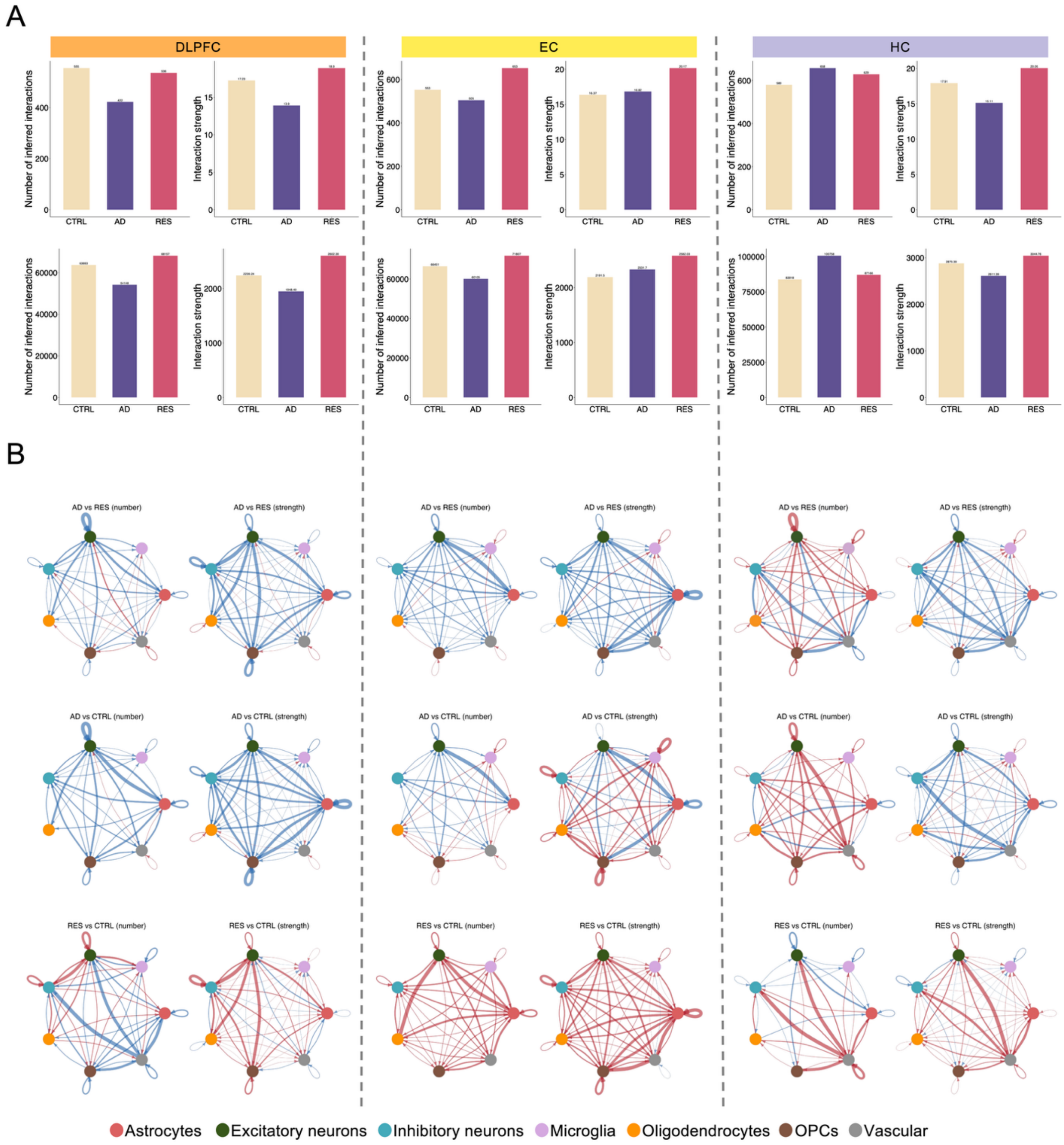

Figure S15. An increase in the number of events in cell-cell communication in resilience. **(A)** Bar plots showing the inferred number and strength of ligand-receptor interactions in each group for major cell populations (top) and cell subpopulations (bottom) in each brain region. **(B)** Differential number and strength of cell-cell interactions for each comparison. Arrows represent direction of interactions, from source to target major cell class. Blue represents a decrease in the number or strength of interactions between any two cells in the first compared to the second diagnostic group, and red represents an increase.
CTRL: Control, AD: Alzheimer's disease, RES: Resilient.

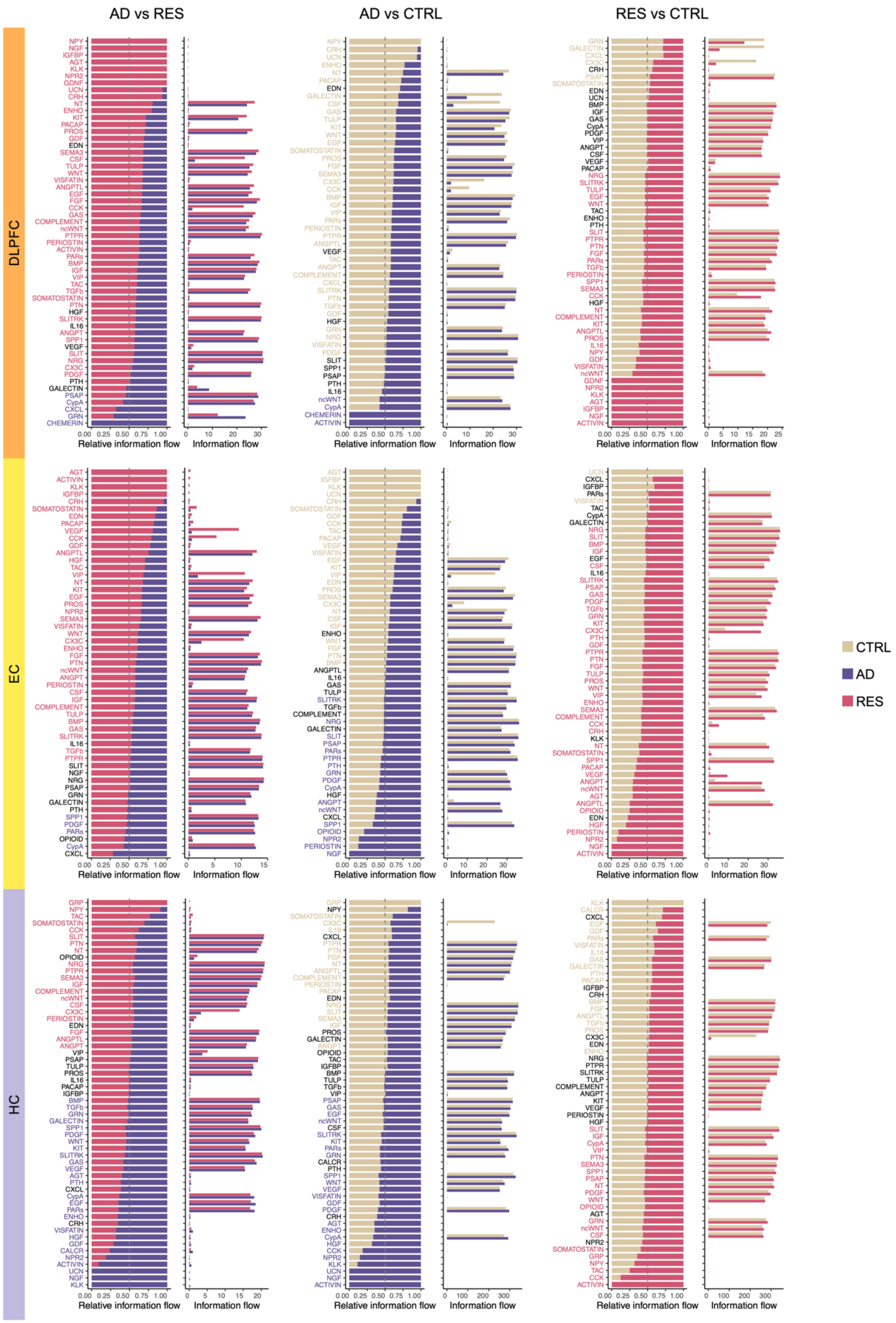
AD vs RES
AD vs CTRL
RES vs CTRL
DLPFC
EC
HC
Relative information flow
Information flow
CTRL
AD
RES

Figure S16. Significant pathways from intercellular communication analysis in cell subpopulations.
List of significant pathways in each brain region (DLPFC, EC, and HC) for each comparison (ADvsRES, ADvsCTRL, and RESvsCTRL). Colored pathway names are significantly shifted towards the group with the corresponding color. Plots on the left show the relative information flow in one diagnostic group compared to another, and the plots on the right show the overall information flow for each signaling pathway.
CTRL: Control, AD: Alzheimer's disease, RES: Resilient.

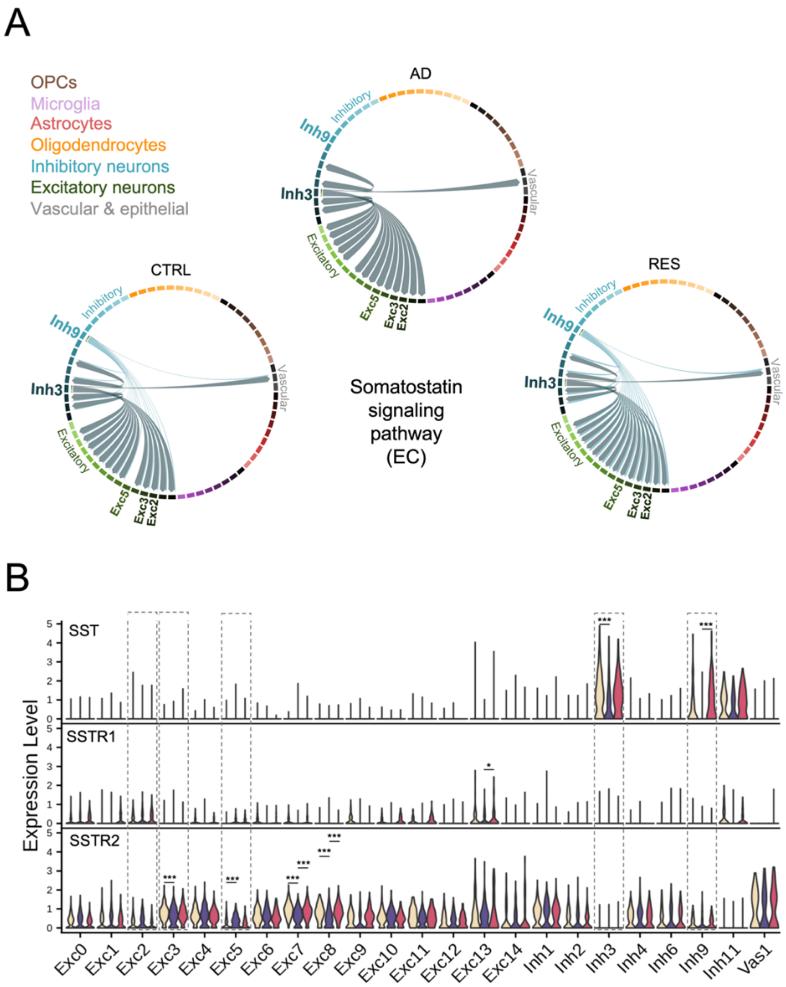

Figure S17. Somatostatin (SST) signaling is dowregulated in AD but not in resilience.
(A) Chord diagrams displaying the SST signaling pathway in cell subpopulations from the EC with significant changes per diagnostic group (**Table S19**).
(B) Expression levels of the ligand and receptors involved in the SST signaling pathway shown in (A). Stars reflect Bonferroni-adjusted P-values < 0.05 (**Table S20**): * adj-P < 0.05, *** adj-P < 0.001.

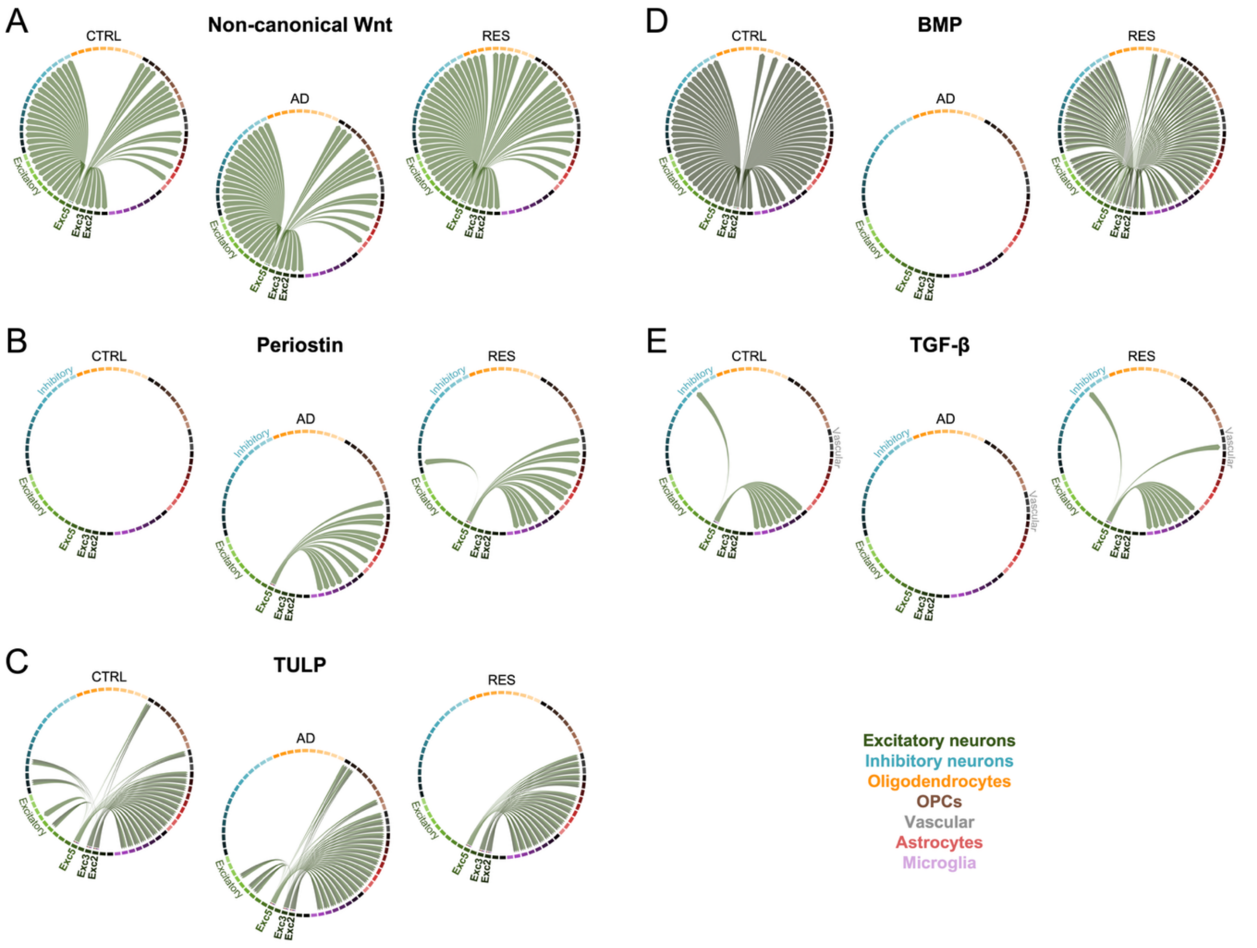

Figure S18. Signaling pathways emerging or disappearing in resilience in excitatory neuronal subpopulations from the entorhinal cortex showing a resilient phenotype. Chord diagrams showing significant networks (Figure S15) having EC:Exc2, EC:Exc:3, or EC:Exc5 as sources.

(A) Non-canonical Wnt (source: EC:Exc5; targets: multiple subtypes of oligodendrocytes; ligand: WNT5A; receptor: MCAM).

(B) Periostin (source: EC:Exc5; target: EC:Inh1; ligand: periostin; receptor: ITGAV/ITGB5).

(C) TULP (sources: EC:Exc2, EC:Exc3, and EC:Exc5; targets: subtypes of excitatory neurons, inhibitory neurons, OPCs, and astrocytes; ligand: TUB; receptor: MERTK).

(D) BMP (source: EC:Exc5; targets: subtypes from all major cell types; ligands: GDF7 and BMP8A; receptors: BMPR1A/ACVR2A, BMPR1A/ACVR2B, BMPR1A/BMPR2, BMPR1B/ACVR2A, BMPR1B/BMPR2, ACVR1/ACVR2A, ACVR1/BMPR2, BMPR1B/ACVR2B).

(E) TGF-β (source: EC:Exc5; target: EC:Fib; ligand: TGF-β2; receptors: TGFBR1/R2 and ACVR1/TGFBR).

CTRL: Control, AD: Alzheimer's disease, RES: Resilient.

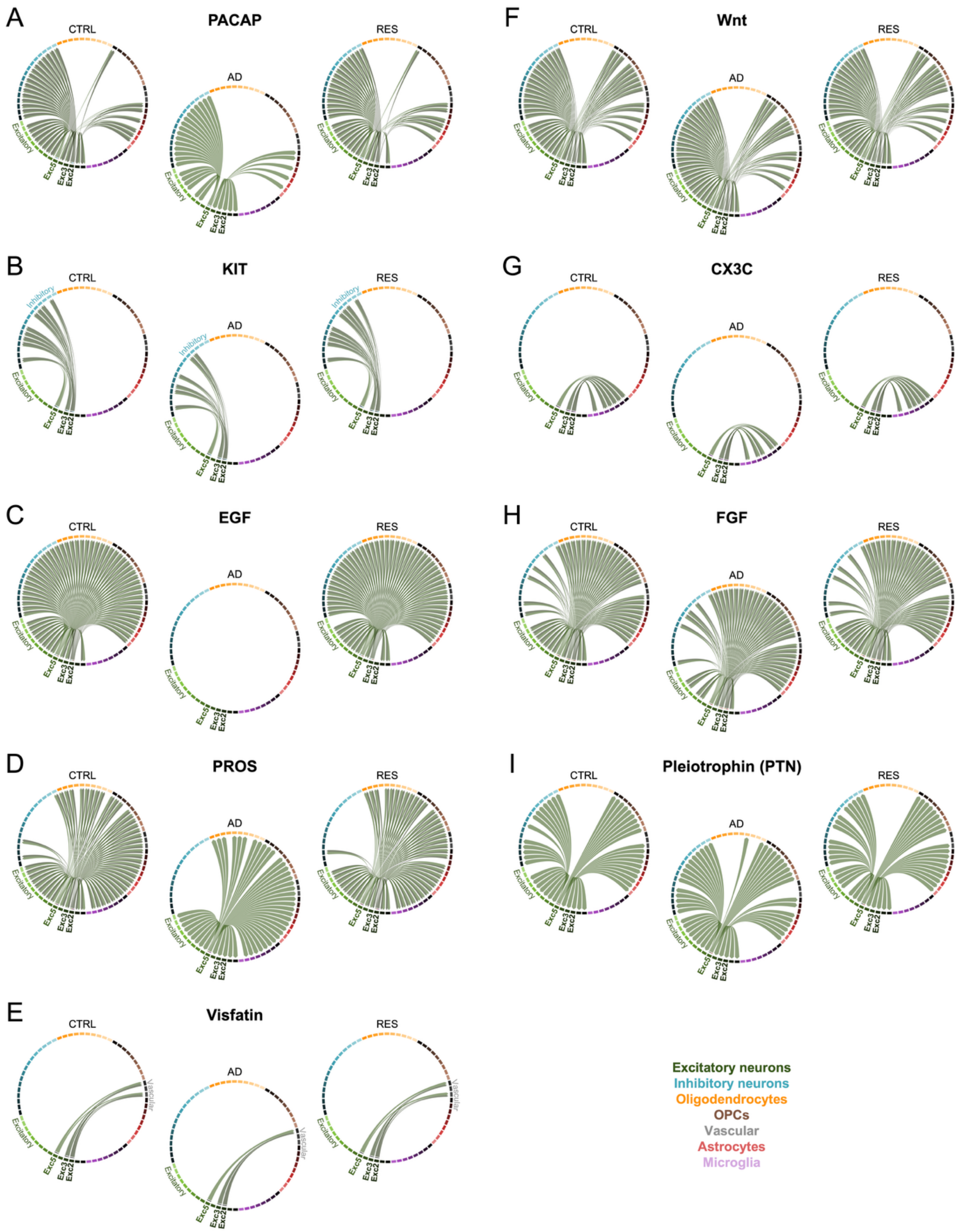

Figure S19. Signaling pathways dynamics in AD in resilience-associated excitatory neuronal subpopulations from the entorhinal cortex.

Chord diagrams showing significant networks (Figure S15) with EC:Exc2, EC:Exc:3, and EC:Exc5 as sources.
(A) PACAP (sources: EC:Exc2 and EC:Exc5; targets: subtypes of multiple major cell types; ligand: ADCYAP1; receptor: ADCYAP1R1).
(B) KIT (sources: EC:Exc2, EC:Exc:3, and EC:Exc5; target: EC:Inh6, EC:Inh10, and EC:Inh14; ligand: KITLG; receptor: KIT).
(C) EGF (source: EC:Exc2 and EC:Exc5; targets: subtypes of all major cell types except microglia; ligands: EGF and BTC; receptors: EGFR and ERBB4).
(D) PROS (source: EC:Exc2; targets: subtypes from all major cell types; ligand: PROS1; receptors: AXL, TYRO3, MERTK).
(E) Visfatin (sources: EC:Exc2, EC:Exc3, EC:Exc5; target: EC:Fib; ligand: NAMPT; receptor:ITGA5/ITGB1).
(F) Wnt (sources: EC:Exc2, EC:Exc3, EC:Exc5; targets: EC:Opc4 (disappears in AD), and EC:Exc14 (emerges in AD); ligands: WNT10B, and WNT3 (in AD only); receptor: FZD3/LRP6).
(G) CX3C (sources: EC:Exc2, EC:Exc3, EC:Exc5; target: EC:Mic2; ligand: CX3CL1; receptor: CX3CR1).
(H) FGF (sources: EC:Exc2, EC:Exc3, EC:Exc5; targets: multiple subtypes of excitatory neurons, inhibitory neurons (loss in some subtypes and gain in others), and EC:Vas0 (emerges in AD); ligands: FGF5, FGF9, and FGF17; receptors: FGFR1 and FGFR2).
(I) Pleiotrophin (source: EC:Exc5; targets: EC:Exc13 (gain), EC:Inh6 and EC:Inh8 (gain), EC:Oli6 and EC:Oli9 (gain), EC:Fib (loss), and EC:Ast2 (loss); ligand: PTN; receptors: PTPRZ1, SDC2 (loss), SDC3, NCL, ALK).
CTRL: Control, AD: Alzheimer’s disease, RES: Resilient.

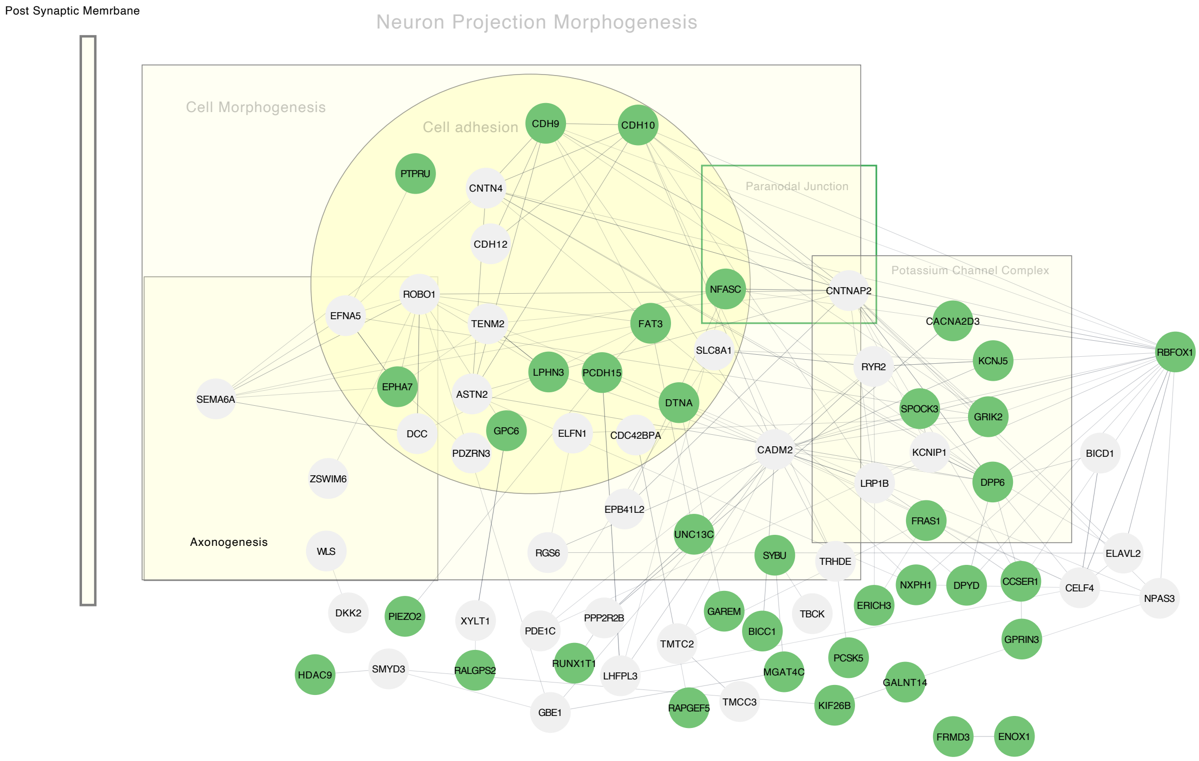

Figure S20. StringDB network of genes associated with risk and protective rare variants also identified as marker genes for DLPFC:Inh1 neurons.
RBFOX1 interaction partners and marker genes are enriched in cell morphogenesis, paranodal junction, potassium channel complex, and axonogenesis. All may contribute to neuron projection morphogenesis. Genes from protective variants are shown as green nodes, and genes from risk variants as gray nodes. Visualization was performed using Cytoscape 3.10.2. (NDEx access to network)

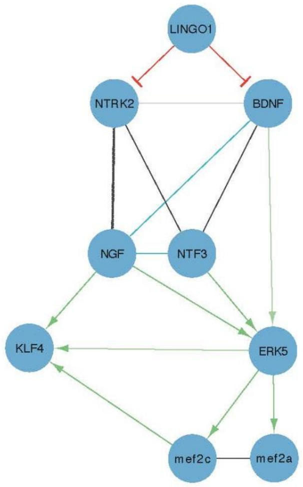

Figure S21. LINGO1 regulation of neurotrophin pathway in resilient cells. STRINGdb relationships and supporting literature were used to establish protein-protein and co-expression relationships between key members of the neurotrophin pathway. Visualization was performed using Cytoscape 3.10.2. Red - inhibition, green arrow, activation. Black, interaction, gray, imputed interaction.

Supplementary references

1. Yen J-HJ, Yu I-CI. The role of ApoE-mediated microglial lipid metabolism in brain aging and disease. Immunometabolism (Cobham). 2023;5:e00018.

2. Yeh FL, Wang Y, Tom I, Gonzalez LC, Sheng M. TREM2 binds to apolipoproteins, including APOE and CLU/APOJ, and thereby facilitates uptake of amyloid-beta by microglia. Neuron. 2016;91:328–40.

3. Tcw J, Qian L, Pipalia NH, Chao MJ, Liang SA, Shi Y, et al. Cholesterol and matrisome pathways dysregulated in astrocytes and microglia. Cell. 2022;185:2213-2233.e25.

4. Barroeta-Espar I, Weinstock LD, Perez-Nievas BG, Meltzer AC, Siao Tick Chong M, Amaral AC, et al. Distinct cytokine profiles in human brains resilient to Alzheimer’s pathology. Neurobiol Dis. 2019;121:327–37.

5. Perez-Nievas BG, Stein TD, Tai H-C, Dols-Icardo O, Scotton TC, Barroeta-Espar I, et al. Dissecting phenotypic traits linked to human resilience to Alzheimer’s pathology. Brain. 2013;136:2510–26.

6. Taddei RN, Perbet R, Mate de Gerando A, Wiedmer AE, Sanchez-Mico M, Connors Stewart T, et al. Tau Oligomer-Containing Synapse Elimination by Microglia and Astrocytes in Alzheimer Disease. JAMA Neurol. 2023. p. 1209–21.

7. Sims R, van der Lee SJ, Naj AC, Bellenguez C, Badarinarayan N, Jakobsdottir J, et al. Rare coding variants in PLCG2, ABI3, and TREM2 implicate microglial-mediated innate immunity in Alzheimer’s disease. Nat Genet. 2017;49:1373–84.

8. Andreone BJ, Przybyla L, Llapashtica C, Rana A, Davis SS, van Lengerich B, et al. Alzheimer’s-associated PLCγ2 is a signaling node required for both TREM2 function and the inflammatory response in human microglia. Nat Neurosci. 2020;23:927–38.

9. Cheung AM-Y, Wang D, Liu K, Hope T, Murray M, Ginty F, et al. Quantitative single-cell analysis of immunofluorescence protein multiplex images illustrates biomarker spatial heterogeneity within breast cancer subtypes. Breast Cancer Res. 2021;23:114.

10. Hao Y, Hao S, Andersen-Nissen E, Mauck WM 3rd, Zheng S, Butler A, et al. Integrated analysis of multimodal single-cell data. Cell. 2021;184:3573-3587.e29.
